# Identification of risk areas and practices for *Taenia saginata* taeniosis/cysticercosis in Ethiopia: a systematic review and meta-analysis

**DOI:** 10.1186/s13071-020-04222-y

**Published:** 2020-07-29

**Authors:** Edilu Jorga, Inge Van Damme, Bizunesh Mideksa, Sarah Gabriël

**Affiliations:** 1 Department of Veterinary Science, College of Agriculture and Veterinary Sciences, Ambo University, P.O. Box 19, Ambo, Ethiopia; 2 Department of Veterinary Public Health, Laboratory of Foodborne Parasitic Zoonoses, Faculty of Veterinary Medicine, Ghent University, Salisburylaan 133, 9820 Merelbeke, Belgium

**Keywords:** Bovine cysticercosis, Ethiopia, Prevalence, Review, Risk factors, Taeniosis, *Taenia saginata*

## Abstract

**Background:**

Bovine cysticercosis (BCC) is an infection of cattle with the metacestode stage of *Taenia saginata*, the beef tapeworm, which causes taeniosis in humans. BCC is responsible for considerable economic losses in the meat sector worldwide. This systematic review and meta-analysis summarizes the prevalence, risk factors and treatment efforts made so far on *T. saginata* infections in Ethiopia, providing a detailed analysis of different factors influencing the varying prevalence estimates in Ethiopia to gain more insight into the occurrence and risk factors of *T. saginata* taeniosis and cysticercosis to date.

**Methods:**

A systematic review and meta-analysis was conducted on data collected from published and grey literature accessed through an electronic database and manual search.

**Results:**

The literature search resulted in 776 outputs of which 132 conformed to the predefined criteria. The average zonal prevalence of meat inspection-based BCC ranged from 2% in Buno-Bedele to 24.6% in Sidama zone. The pooled prevalence of BCC was influenced by the number of muscle/organs inspected, ranging from 3.4% (95% CI: 1.7–5.1%) using fewer predilection sites to 19.4% (95% CI: 13.3–25.4%) using inspection of a maximum number of predilection sites. None of the tested variables were significantly associated with BCC. Questionnaire-based taeniosis ranged between 19.0% in Halaba special woreda to 70.0% in Gedeo zone and stool test-based taeniosis varied from 0.6% in central Tigray to 10.7% in Gurage zone. Questionnaire-based prevalence of taeniosis was higher in people with a frequent raw beef consumption habit (pooled OR, pOR: 10.5, 95% CI: 6.0–17.9), adults (pOR: 2.5, 95% CI: 1.7–3.6), men (pOR: 2.8, 95% CI: 2.1–3.6), and Christians (pOR: 2.0, 95% CI: 1.4–2.8) compared to less frequent raw beef consumers, younger people, women and Muslims, respectively.

**Conclusions:**

This review revealed a widespread but variable occurrence of BCC and taeniosis in Ethiopian regions and zones, urging for harmonized and enhanced detection for improved control of the parasite. Accurate prevalence estimates using more sensitive tests, detailed risk factor analysis, as well as data on financial losses are needed to develop effective control strategies for the Ethiopian epidemiologic condition.
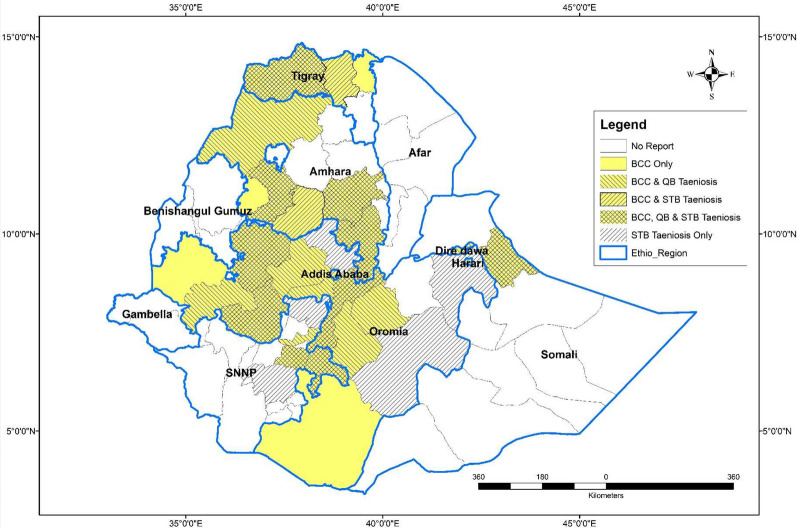

## Background

Bovine cysticercosis (BCC) is an infection of cattle with the metacestode stage of the tapeworm *Taenia saginata* [1]. Despite its global distribution, the highest numbers of tapeworm carriers are observed within communities in developing countries. However, due to the limited public health impact of taeniosis, lack of data on the economic impact of BCC and taeniosis, the existence of other priority diseases and limited resources, *T. saginata* taeniosis/cysticercosis remains a neglected zoonosis [2, 3].

The definitive host (human) becomes infected with *T. saginata* by ingestion of viable cysticerci in raw or undercooked beef. In the intestine, the adult worm stage measures 4–12 meters in length and individuals may remain infected for several years. About 6–9 proglottids are shed daily, either on defecation or by active migration. Each proglottid contains 50,000–80,000 eggs [4], and up to 720,000 *T. saginata* eggs can be released daily into the environment by a single infected human. The life-cycle is maintained when infected people contaminate the environment/animal directly (as a result of open defecation, active migration of the proglottids into the environment (including feed) or unhygienic practices leading to contamination/infection *via* hands), or indirectly *via* urban sewage effluent [5].

Following ingestion of the eggs with contaminated feed, fodder or water by the intermediate host (cattle), the oncosphere penetrates the intestinal wall to reach the skeletal and cardiac muscles and other tissues, where they develop into cysticerci (BCC) and become infective to humans after 10 weeks [4]. The cysticerci in the striated muscles start to degenerate and calcify within a few months following infection, and after 9 months the number of viable cysticerci is reduced substantially [3].

Human taeniosis is associated with minor abdominal discomfort, nausea, mild diarrhoea, weight loss, and anal pruritus, though serious digestive disorders such as intestinal blockage or perforation and peritonitis have been reported [6, 7]. The clinical effect of BCC is generally insignificant in natural infections, but it accounts for considerable economic losses to the food industry due to condemnation, freezing and downgrading of infected carcasses [5].

Diagnosis and control of BCC is primarily based on meat inspection, which involves inspection for cystic lesions using palpation and incision of defined muscles, although the adopted inspection techniques and the final judgments vary greatly throughout the world [3]. Routine meat inspection generally has a low sensitivity (< 15%), especially so for low levels of infection as estimated recently in Belgium (0.76%) [8, 9]. As an alternative, immuno-diagnostic tools such as enzyme-linked immunosorbent assays (ELISAs) detecting either specific antibodies or circulating antigens have been developed for BCC [10, 11]. While Ogunremi and Benjamin [11] estimated the sensitivity and specificity of their antibody detecting ELISA (based on excretory secretory antigen) at 92.9% and 90.6%, respectively, this could not be confirmed in a later study, where a sensitivity of 13.8% and a specificity of 92.9% was estimated in animals with low levels of infection [8]. The genus-specific antigen detection ELISA has a high sensitivity (98.7%) for cattle infected with more than 50 viable cysticerci, but the sensitivity reduces significantly when less than 50 cysticerci are present (12.8%) [8, 9]. For the detection of human taeniosis cases, the routinely used coprological techniques are also known to have low sensitivities [12] and lack species-specificity. Several copro-Ag ELISAs have been developed to detect *Taenia* spp. antigens in human stool samples [13–15]. Although these tests were originally developed for the detection of *T. solium*, most are genus-specific and can thus be used for *T. saginata* taeniosis as well, when further species identification by molecular tools is conducted. Copro PCR assays for direct detection in stool samples have been developed [16, 17], and different PCR-based tests are available for the identification of proglottids or suspected cysticerci to *Taenia* species level, including PCR-restricted fragment length polymorphism (RFLP) and multiplex PCR [16, 18, 19]. However, these ELISAs and PCR assays are not commercially available and are not routinely used.

Over the last decades, several studies on *T. saginata* cysticercosis and taeniosis have been conducted in different regions in Ethiopia, applying different sampling and often poor diagnostic methodologies, resulting in varying outcomes. Prevalence estimates based on meat inspection have been reported between 1.2% [20] and 32.2% [21] and prevalence estimates of questionnaire-based human taeniosis have been described between 19.0% [22] and 82.6% [23]. Recently Dermauw et al. [24] reviewed the distribution of *T. saginata* taeniosis/cysticercosis in Eastern and Southern Africa with an inter-country context. Hiko and Seifu [25] also gave an overview of different reports in Ethiopia.

In this systematic review, rather than a descriptive presentation of occurrence, a more detailed analysis of different factors influencing the varying prevalence estimates in Ethiopia was undertaken to gain more insight into the occurrence of *T. saginata* taeniosis and cysticercosis to date. With regard to the variation among the prevalence reports, different factors, such as the study set-up, study population, sample size, host and environmental factors, and the diagnostic strategy may all affect prevalence estimates. Therefore, the aim of this systematic review and meta-analysis was to present a qualitative and quantitative summary on the prevalence, distribution and risk factors of *T. saginata* taeniosis/cysticercosis in Ethiopia based on the existing literature. Additionally, data on carcass condemnation, drug inventory records and taenicidal herbs were summarized from the retrieved manuscripts.

## Methods

### Systematic review protocol

**Fig. 1:**
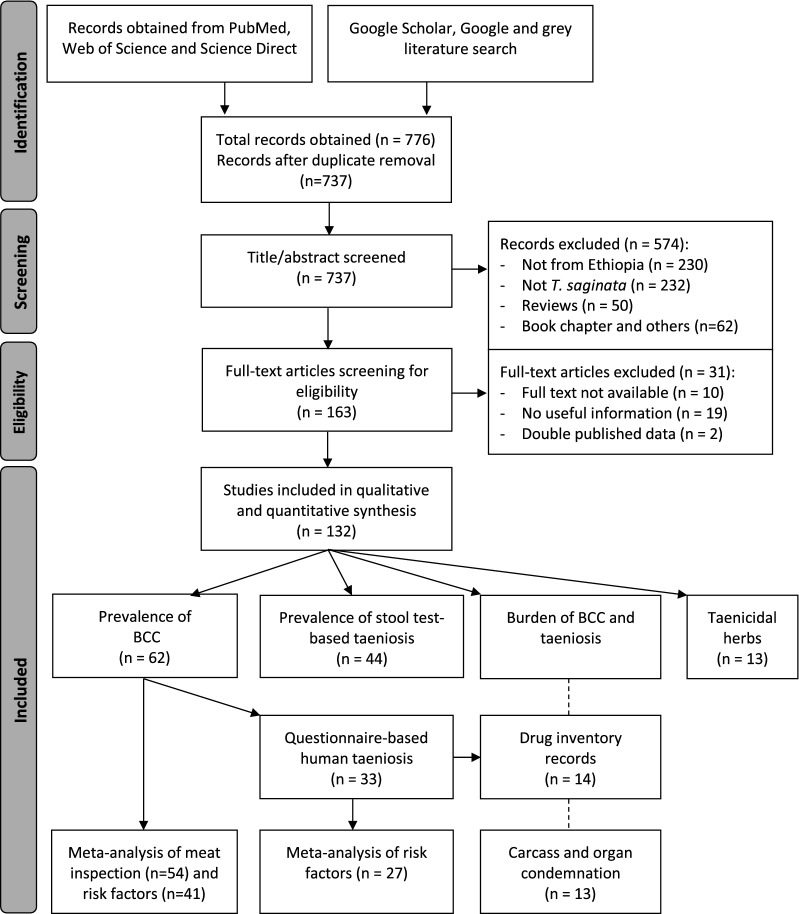
Flow chart describing the output of the literature search and selection of articles

The review question was as follows: ‘What are the prevalence, distribution, and risk factors for BCC and taeniosis in Ethiopia and which taenicidal herbs are used in Ethiopia?’. This research question enabled defining the inclusion criteria, developing the search strategy and data collection. The approach for the review protocol followed the principles of the PRISMA guidelines for systematic reviews [26] (Additional file 1: Table S1). An overview of the literature searches and selection process is shown in Fig. 1.

### Search strategies

The search strategy includes the following databases: PubMed, ScienceDirect and Web of Science and a further search was carried out using Google Scholar and Google as well. An advanced search was followed and the search terms and Boolean operation combination used were as follows: For PubMed: (“Bovine cysticercosis” OR “*Cysticercus bovis*” OR Cysticercosis OR “*C. bovis*” OR “Metacestode” OR “*Taenia saginata*” OR “*T. saginata*” OR “*Taenia* spp.” OR “*Taenia* species” OR “Taeniasis” OR “Taeniosis”) AND “Ethiopia”. For Web of Science: (“Ethiopia” AND (“Bovine cysticercosis” OR “*Cysticercus bovis*” OR “Cysticercosis” OR “*C. bovis*” OR “Metacestode” OR “*Taenia saginata*” OR “*T. saginata*” OR “*Taenia* spp.” OR “*Taenia* species” OR “Taeniasis” OR “Taeniosis) and for ScienceDirect “Bovine cysticercosis” OR “*Cysticercus bovis*” OR “*C. bovis*” OR “*Taenia saginata*” OR “*T. saginata*” OR “*Taenia* species” OR “*Taenia* spp.” OR “Taeniosis” OR “Taeniasis” AND “Ethiopia”. For ScienceDirect, the search was restricted to the title, abstract or author-specified keywords. In addition, the references cited in the published manuscripts were cross-checked to capture any relevant reports that might have been missed in the electronic search process. Similarly, Google and Google Scholar were used to search articles published in local journals such as the Ethiopian Veterinary Journal/Proceeding and Bulletin of Animal Health and Production in Africa and grey literature. Additional searches for grey literature such as MSc thesis reports, were done at the repositories of universities and research centres of Ethiopia and by contacting the original authors.

### Inclusion and exclusion criteria

Literature reporting the prevalence and/or risk factors of BCC and/or human taeniosis, taenicidal reports including relevant information on economic loss estimation from Ethiopia were all included. The results of the search were not restricted by year of publication (until 27 March 2019), study design, journal, or status of publication. Review papers, book chapters, letters to the editor and editorials without original data and articles whose full text was not available or with insufficient information in the abstract were excluded.

### Study selection

Search results were combined to manage duplicates. Then a selection was made based on the title and abstract screening, followed by a full text reviewing. Articles were organized and grouped into five different topics as follows: (i) prevalence of BCC; (ii) prevalence of taeniosis, based on stool tests and questionnaires; (iii) organ and carcass condemnation reports; (iv) drug inventory reports; and (v) taenicidal and herbal research reports.

### Data extraction

Data from selected articles were recorded in Excel sheets for each of the topics. The following information was recorded for studies regarding the prevalence of BCC: author names and year of publication; journal name and volume; data period; study region; study subjects; study objective; study setup; diagnostic method used; number of study sites/abattoirs; sample size; number of positive animals, muscles and organs where cysts were detected; total cysts collected; and proportion of viable cysts. Similarly, for data regarding questionnaire- and stool test-based human taeniosis, author names and year of publication, journal name and volume, data period, study region, study objective, study set-up, diagnostic method used, sample size, and number of positives were recorded. For the evaluation of risk factors, the sample size, the number of positives and negatives in each category and the cut-off value points for variables such as age were recorded. Some variables were categorised in different levels by the different authors (such as occupation and educational status) and were re-grouped for the meta-analysis. The total number of carcasses and organs condemned and condemnation due to BCC with the estimated cost was recorded. The reported adult taenicidal dosages sold and the estimated cost were also recorded. Lastly, information such as study region, study setup, study period, taenicidal plant species and anthelmintic drug type used were described.

### Data analyses

Prevalence reports for BCC from the same zonal area were converted to a zonal prevalence. An average zonal prevalence was preferred over a country-level estimate to account for potential regional differences in the prevalence. The synthesized results were presented by maps using ArcGIS 10.4 (ArcGIS Inc., New York, USA), to visualize the zonal and regional distribution of taeniosis/cysticercosis in Ethiopia. In addition, data on zonal cattle population of Ethiopia obtained from CSA 2014/2015 was converted to a map [27]. Similarly, a map showing the sanitary status and possibility of open defecation in the country was obtained from World Bank report [28].

The location of cysticerci in carcasses/predilection sites was recorded as the proportion of positive organs relative to the total number of positive cattle and the percentage of viable cysts relative to the total number of cysts collected.

Meta-analysis was performed using STATA version 14.0 (StataCorp 4905 Lakeway Drive College Station, Texas 77845, USA). The study level prevalence was transformed to logit event estimate and the corresponding variances were calculated. The random effect model was used to pool the logit event estimates and later the pooled logit estimates were back-transformed to prevalence estimates i.e. the pooled prevalence. To evaluate the effect of the number of organs/muscles that are inspected on BCC prevalence, a pooled prevalence was estimated for different subgroups. In most Ethiopian abattoirs the routine meat inspection regulation is not strictly followed. As a result, there is variation in the number of muscles/organs that are inspected. The most frequently inspected muscles/organs are the shoulder muscle, heart masseter and tongue. Due to this variation, studies were categorized in four different groups: (i) studies that included at least the following 8 organs/muscles: shoulder, heart, masseter, tongue, diaphragm, thigh muscle, liver and intercostal muscle; (ii) studies that included at least the following 6 organs/muscles: shoulder, masseter, tongue, heart, liver and diaphragm/lung or studies that inspected at least seven organs/tissues in total; (iii) studies that included at least 5 of the following organs/muscles: shoulder, masseter, tongue, heart and liver or diaphragm/thigh or at least six organs in total; and (iv) studies that include at most 4 of the following organs/muscles including: shoulder, masseter, tongue, heart or other organs/muscles. For stool test-based human taeniosis, subgroup analysis was done for the different study populations, so studies were categorized in the following groups: (i) general community; (ii) food handlers; (iii) patients; and (iv) school children. Risk factor analysis was performed for BCC and questionnaire-based taeniosis using random effect models to pool the effect sizes of the individual studies. For each variable considered, the pooled odds ratio (OR) and its 95% confidence interval (CI) were calculated. The heterogeneity between studies was assessed by Cochran’s Q test and the percentage of the variation in the estimates attributable to heterogeneity was quantified by the inverse variance index (I^2^) [29]. Higher values of I^2^ signify a greater degree of variation.

## Results

### Literature search result

**Fig. 2:**
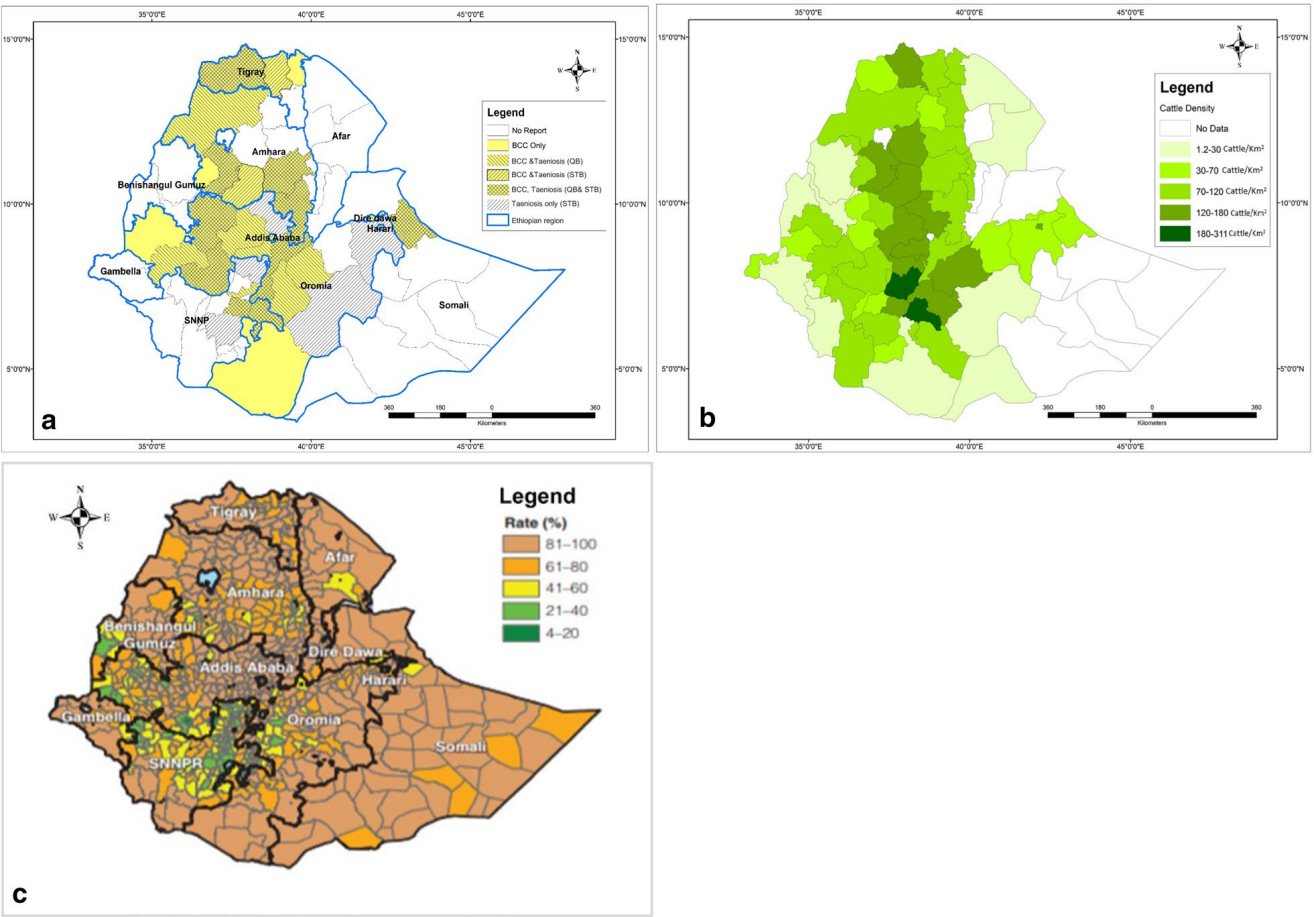
Map of Ethiopia showing regions and zones where **a** bovine cysticercosis (BCC), taeniosis (questionnaire-based (QB)), and taeniosis (stool test-based (STB)) is reported. **b** Cattle population density in Ethiopia (expressed as the number of cattle per km^2^). **c** The open defecation status in Ethiopia (expressed as the proportion of people without a latrine). Copyright: Creative Commons Attribution license (CC BY 3.0 IGO). Citation: World Bank. 2018. Maintaining the Momentum while Addressing Service Quality and Equity: A Diagnostic of Water Supply, Sanitation, Hygiene, and Poverty in Ethiopia. WASH Poverty Diagnostic. World Bank, Washington, DC [28]

A total of 776 articles written in English were obtained from all data sources (including eight from the additional search), of which 39 were duplicates and 574 excluded as irrelevant based on the criteria following the screening of titles and abstracts (Fig. 1). One hundred sixty-three were passed for full article reading, out of which 31 were excluded, two of which were duplicate data. One hundred and thirty-two papers met the predefined criteria to be included. They were grouped as BCC prevalence (*n* = 62), stool test-based prevalence of taeniosis (*n* = 44), questionnaire-based taeniosis (*n* = 33), organ and carcass condemnation (*n* = 13), drug inventory records (*n* = 14) and taenicidal studies (*n* = 13). Sixty-one papers were used for zonal prevalence averaging of BCC, of which 54 (two articles reporting from two different regions were considered as each time two studies; *n* = 56) were selected for meat inspection-based meta-analysis of BCC and 41 of them were included for at least one BCC risk factor analysis. Of the BCC prevalence studies, 33 reported questionnaire-based prevalence of taeniosis and 14 included drug inventory records. An overview of the different regions and zones in Ethiopia where BCC and/or taeniosis was reported is shown in Fig. 2a.

### Prevalence of BCC

Out of the 62 articles, 61 provided information on meat inspection-based prevalence of BCC, and the remaining one was a molecular study. Ethiopia has 9 regions and two chartered cities (Addis Ababa and Dire Dawa) and BCC reports were identified from 6 regions and the two chartered cities i.e. all except Gambela, Benishahgul Gumuz and Afar regions (Fig. 2a). The majority of the reports were from Oromia region (central Ethiopia), Amhara region and parts of the Southern Nations and Nationalities People region (SNNP). Within these regions, BCC was reported in 27 different zones. Reports were obtained from 46 abattoirs; 42 were municipal abattoirs slaughtering ruminants for local consumption and 4 were export abattoirs. Twelve reports were from East Shoa zone of Oromia region. The maximum number of reports per abattoir was six for Gondar (prevalence estimates range of 2.0–18.0%), five for Addis Ababa (1.9–7.5%) and Jimma (2.9–5.1%). One seroprevalence report (25.6%) was obtained from Addis Ababa abattoir using an indirect hemagglutination test (IHAT) [30]. One molecular study from eastern and central Ethiopia identified that 92.7% (38/41) of the cysticerci from bovine carcasses were *T. saginata*, whereas 7.3% (3/41) of them were suggested to be *Taenia* spp. from wildlife (*T. hyaena*) [31] (Additional file 2: Table S2).

**Fig. 3:**
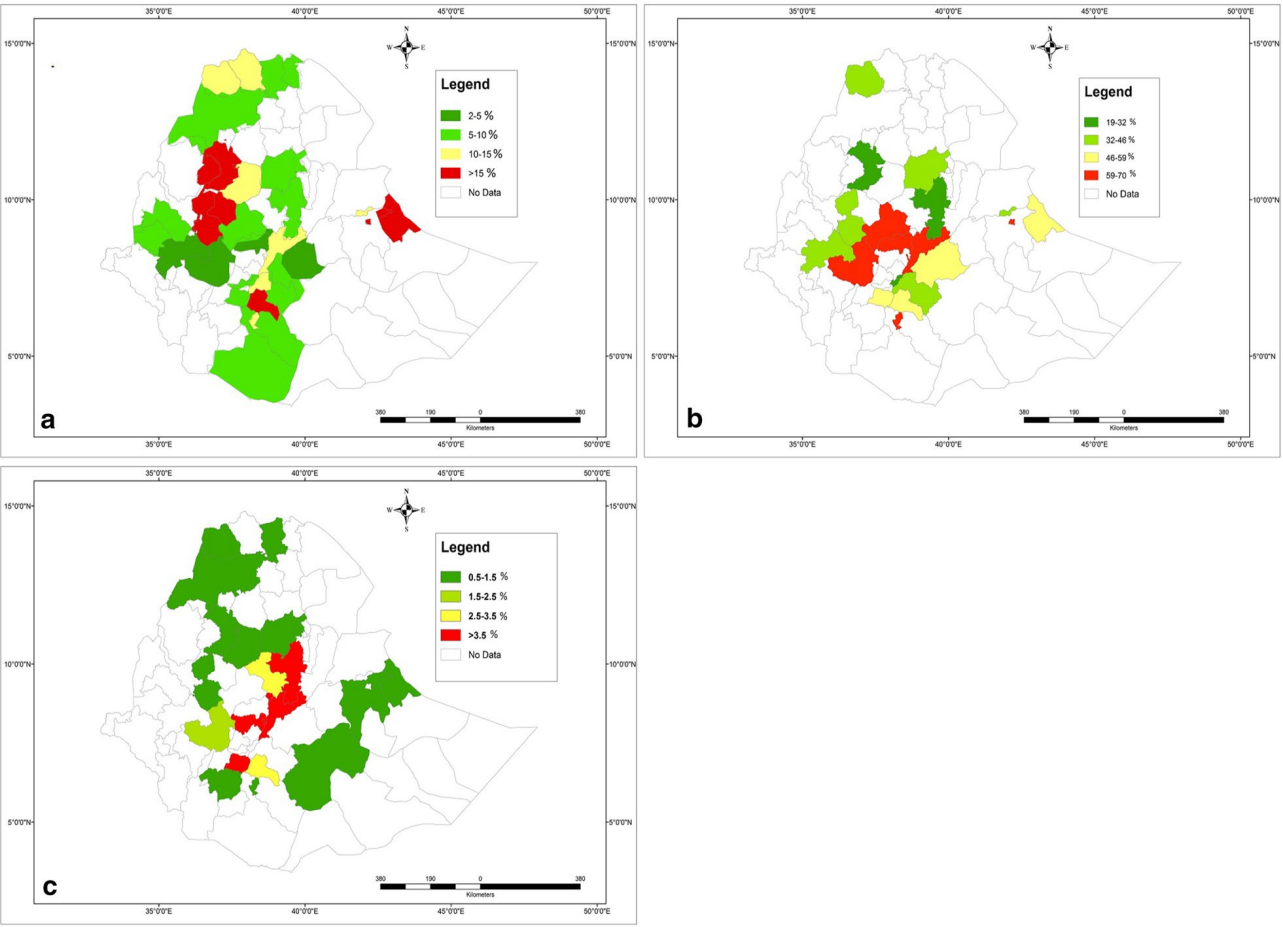
Map of Ethiopia displaying the zonal prevalence of **a** bovine cysticercosis (BCC), **b** taeniosis (questionnaire-based) and **c** taeniosis (stool test-based)

**Fig. 4:**
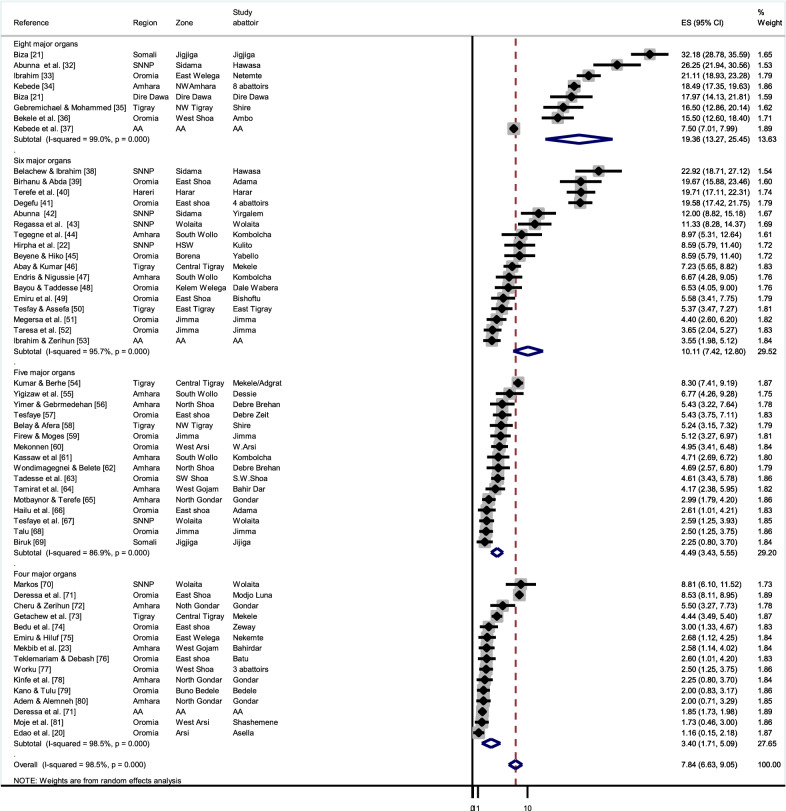
Forest plot showing an overview of studies reporting bovine cysticercosis (BCC) in Ethiopia, grouped by the number of organs/muscles that were inspected during *post-mortem* meat inspection. The box size shows the weight of the study and the middle of the box (dot) represents the point estimate of the study, the length of the horizontal lines indicates the 95% CI; the vertical broken line indicates the pooled estimate; the diamond-shaped box at the bottom represents the 95% CI; the solid line indicates the point of null assumption

The averaged zonal prevalence of BCC was maximum in Sidama zone, SNNP (24.6%) and the lowest in Buno Bedele zone, western Ethiopia (2.0%) (Fig. 3a). The overall pooled prevalence estimate of BCC in Ethiopia was 7.8% (95% CI: 6.63–9.05%). The calculated Cochran Chi-square value (Q) of 3783 (*df* = 55, *P* < 0.001) and the inverse variance index value (I^2^) of 98.5% indicates a high degree of heterogeneity among the reports. Since the methodology of meat inspection varied among the reports, subgroup analysis was performed depending on the number of organs/tissues that were inspected. The pooled prevalence estimate was 19.4% (95% CI: 13.27–25.45%, *df* = 7) for studies inspecting eight or more muscles/organs, whereas it was the 3.4% when 4 or less muscles/organs were inspected (95% CI: 1.71–5.09%, *df* = 14) (Fig. 4; [32–81]). Nevertheless, the high I^2^ values (> 85%) for each of the subgroups indicate a high degree of heterogeneity between studies applying a similar methodology.

The location of the cysticerci in predilection sites was reported in 54 articles (Additional file 2: Table S3). The heart and tongue were the most frequently inspected organs and were excluded from meat inspection in only one report each. The masseter muscle, shoulder muscle, and liver were not included by 3, 8, and 14 studies, respectively. The neck muscle and longissimus dorsi muscle were inspected by only 3 and 2 reports, respectively, and the internal organs (kidney, spleen, intestinal mucosa) were inspected in less than 10 reports. In the 6481 infected cattle, 9935 organs were found infected and cysticerci were mostly found in the tongue (24.2%), heart (23.2%), shoulder muscle (18.8%) and masseter muscle (14.5%). The detection rate was lower in the remaining predilection sites and the lowest was recorded in the intestinal mucosa and hump (0.02%). The proportion of viable cysticerci among the cysticerci collected was 55.8% (3523/6309) (Additional file 2: Table S4).

### Meta-analysis for risk factors of BCC

**Fig. 5:**
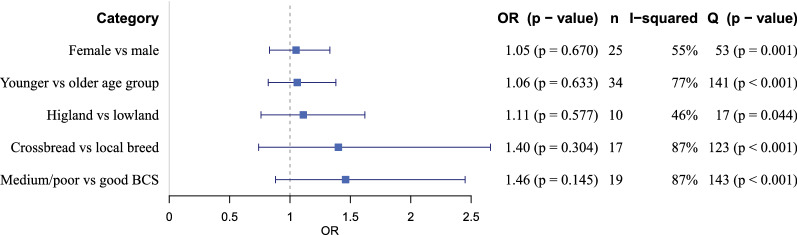
Overview of the meta-analyses results for different risk factors for bovine cysticercosis (BCC) in Ethiopia. *Abbreviations*: OR, pooled odds ratio; n, number of studies that are included in the analysis; I^2^, inverse variance index representing the percentage of the variation in the estimates attributable to heterogeneity; Q, Cochrans Q test value representing heterogeneities of the study level estimates

Forty-one articles reporting risk factor for BCC were included for meta-analysis of at least one variable. Sex, age, breed and body condition score (poor/medium or good) of the slaughtered cattle and altitude (highland or lowland) from where the animals were brought were the variables analysed. The summary of the relationship between the studied variables and BCC is shown in Fig. 5. The reports were heterogenic for all the variables that were tested (I^2^ > 45%). However, the calculated odds ratios showing the risk of exposure to BCC did not differ significantly for any of the variables that were tested. The highest OR was obtained for cattle of medium/poor condition having a 1.46 (pooled OR: 1.46, 95% CI: 0.88–2.45, *P* = 0.145) higher odds for BCC infection as compared to animals with a good body condition.

### Prevalence of taeniosis

Thirty-three of the bovine cysticercosis studies reported also a questionnaire-based prevalence of human taeniosis. In all the reports, ‘having seen the proglottids in the stool and underwear’ was considered as a positive finding. The highest average zonal prevalence was 70.0% obtained at Yirgalem, SNNP region of Ethiopia (Fig. 3b, Additional file 2: Table S5). Reinfection of up to two to six times per year was also reported [32, 41]. The country-wide pooled prevalence for questionnaire-based taeniosis was 52.3% (95% CI: 46.4–58.2%, *df* = 32), and the Q and I^2^ values (Q = 719.7 and I^2^ = 95.6%, *P* < 0.001) are indicative of heterogeneity among the reports (Additional file 3: Figure S1).

**Fig. 6:**
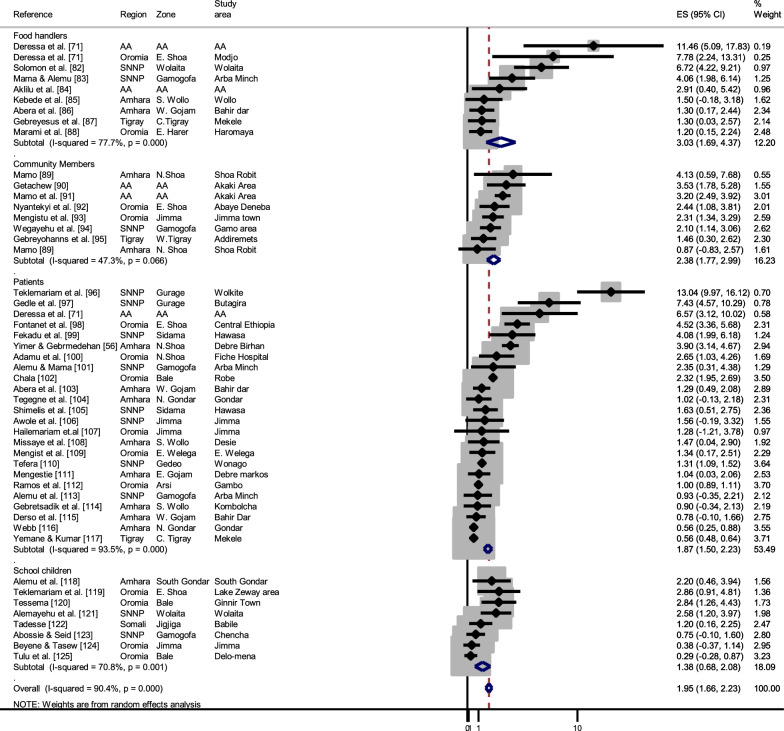
Forest plot showing an overview of studies reporting stool test-based human taeniosis prevalence in Ethiopia, grouped per study population. The box size shows the weight of the study and the middle of the box (dot) represents the point estimate of the study, the length of the horizontal lines indicates the 95% CI; the vertical broken line indicates the pooled estimate; the diamond-shaped box at the bottom represents the 95% CI; the solid line indicates the point of null assumption

Forty-four articles were obtained reporting human intestinal parasitic infections based on stool tests. Only one report was exclusively about taeniosis [71], whereas the remaining papers reported taeniosis together with other intestinal parasites. The overall stool test-based pooled prevalence of taeniosis in Ethiopia was 1.9% (95% CI: 1.6–2.2%, *df* = 43). Eight of the reports were based on a sampling of the general community, whereas the remaining papers targeted specific groups, such as hospital patients, food handlers and school children. The formol-ether concentration technique was most frequently used for detection of *Taenia* eggs, Kato-Katz and Modified Ziehl Neelsen staining techniques were also used. The average zonal prevalence shows that taeniosis is more common in central Ethiopia and parts of the Southern region (Fig. 3c, Additional file 2: Table S6). Region-based grouping showed 4.1% pooled prevalence at Addis Ababa (95% CI: 2.6–5.6%) and 3.2% at SNNP (95% CI: 2.2–4.2%). Study population-based subgroup analysis showed that the pooled prevalence was 3.0% in food handlers (95% CI: 1.7–4.4%), followed by 2.4% the general community (95% CI: 1.8–3.0%), 1.8% in hospital patients (95% CI: 1.4–2.1%) and it was 1.3% in school children (95% CI: 0.6–2.0%) (Fig. 6; [82–125]). The reports within each of the subgroups of patients were highly heterogeneous (I^2^ > 90%).

### Meta-analysis of risk factors for questionnaire-based taeniosis

**Fig. 7:**
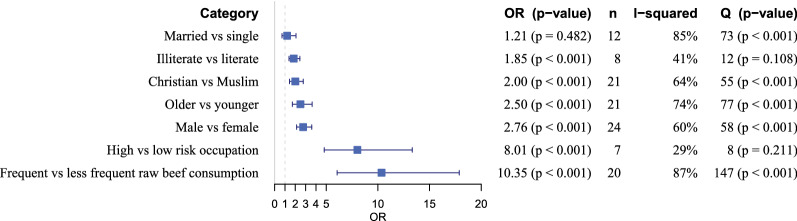
Overview of the random effects meta-analyses results for different risk factors for questionnaire based taeniosis in Ethiopia. *Abbreviations*: OR, pooled odds ratio; n, number of studies that are included in the analysis; I^2^, inverse variance index representing the percentage of the variation in the estimates attributable to heterogeneity; Q, Cochrans Q test value representing heterogeneities of the study level estimates

Among the 16 studied variables, only 7 were included for risk factor analysis for the questionnaire-based human taeniosis. The remaining variables were reported by less than four researchers, such as knowledge about *T. saginata*, use of latrines, residential area, use of spices in raw beef, raw beef preparation, community type, income, meat source and drug use; hence no clear trends were obtained for these variables (data not shown). For educational status and occupational status, seven and four reports respectively, were omitted from the meta-analysis due to either unclear cut-off values or too diverse categories. Because of the latter reason, educational status was thus limited to literacy (illiterate *versus* literate) only. Gender, religion, raw beef consumption habit, age, occupational group, literacy, and marital status were included in the final analyses (Fig. 7). The Cochran Q and I^2^ values showed that studies for all variables except occupation and literacy were heterogeneous. All the variables except marital status showed a significant association with taeniosis infection. Hence, males (pOR: 2.76, 95% CI: 2.13–3.59; *P* < 0.001), Christians (pOR: 2.00, 95% CI: 1.44–2.77), raw beef consumers (pOR: 10.35, 95% CI: 6.05–17.87), and older age groups (pOR: 2.50, 95% CI: 1.72–3.63) had a higher odd of self-reported taeniosis than females, Muslims, less frequent raw beef consumers and younger age groups, respectively.

### The economic losses of *Taenia saginata* infection

Little information is available on the costs related to *T. saginata* in Ethiopia. Taeniosis patients are unlikely to seek health care unless the clinical signs and symptoms are very serious. *Taenia saginata* infection might be diagnosed while patients are visiting health institutions for other intestinal illnesses in which case they are treated based on the physician’s prescription. In most cases, it is a common practice to purchase taenicides from a pharmacy following the finding of proglottids in the stool (self-diagnosis).

Fourteen reports were obtained regarding pharmacy inventory, originating from nine zones of Ethiopia. The number of pharmacies that were included was not specified in five of the reports and in all the reports information was obtained from voluntary pharmacies only. Niclosamide and mebendazole were the most frequently sold drugs, followed by albendazole and praziquantel. The maximum number of adult taenicidal doses sold was 472,013 doses over a 5-year period at Hawasa, southern Ethiopia [42], the highest estimated taenicidal cost was US$ 93,310 during four years from seven pharmacy shops at Wolaita Sodo, southern Ethiopia [43] (Additional file 2: Table S7).

Thirteen meat inspection-based studies reported organ and carcass condemnation from different abattoirs of the country. According to these reports, the tongue, heart and carcass were most frequently condemned due to BCC. About 37.5% of the total condemned tongues were reported due to BCC, followed by the heart (15.1%) and carcass (5.3%). One study from the Tigray region, northern Ethiopia estimated €2402.4 loss from carcasses and organ condemnation due to BCC within six-month time from three abattoirs, where the prevalence of BCC was 8.3% (308/3711) [54] (Additional file 2: Table S8).

### Taenicidal treatment

Thirteen articles were obtained reporting either herbal taenicides or anthelmintic efficacy study. The Ethiopian indigenous tree *Hagenica abyssinica*, locally named kosso has been used traditionally as a remedy against *T. saginata* infection before it was discovered by Europeans in the early seventeenth century [126]. Several plants were reported to be used by Amhara, Shinahsa and Agew-Awi ethnic groups in north-western Ethiopia [127]; Meinit people [128] and Sheko people [129] in south-western Ethiopia. Some authors reported frequently used herbs in central Ethiopia [66, 77, 130]. Generally, *Hagenia abyssinica*, *Cucurbita pepo*, *Embelia schimperi*, *Glinus lotoides* and *Myrsine africana* were the most frequently reported taenicidal plants in Ethiopia. Surveys showed that about 10–15% of people living in urban areas are still using traditional remedies for taeniosis [51, 66, 74, 76]. However, only a few in vitro trials focusing on potency and the toxicity margin of some of these medicinal plants were done. For instance, the in vivo trial on mice with crude hydroalcoholic extract of *E. schimperi* at 1000 mg/kg dose showed 100% clearance of the parasite *Hymenolepis nana* [131]. Desta [132] ranked 10 commonly used taenicidal herbs on the basis of lower toxicity, higher potency and shorter worm expulsion time. The top three were *Echinops giganteus*, *Embelia schimperi*, *Hagenia abyssinica*, with their medical effective dose in gram and worm expulsion time in hours of 7.84 ± 1.04 and 10.2 ± 2.0; 8.23 ± 1.50 and 10.8 ± 1.0; and 12.5 ± 2.2 and 11.3 ± 1.4, respectively. Demma et al. [133] studied the toxicity margin of *Glinus lotoides* seeds and found it is safe at repeated doses. Past studies in Ethiopia showed that praziquantel was effective against *T. saginata* at a single oral dose (10 mg/kg body weight), whereas clinical and parasitological cures were obtained by a regimen of 2 g of niclosamide given on three consecutive days [134, 135].

## Discussion

*Taenia saginata* taeniosis and cysticercosis are highly endemic in central and eastern African countries such as Ethiopia [3]. In this review, we observed a high prevalence of BCC recorded in several studies, with substantial variation in the prevalence among the different zones of the country and within the same abattoir at different time points. The within-abattoir difference could be primarily attributed to variations in the application of the Ethiopian meat inspection regulation. The Ethiopian Meat Inspection Regulation (1972) [136] recommends visual inspection and palpation of all exposed surfaces, followed by incisions in the heart, triceps muscles, internal and external masseter muscle, tongue, the thigh muscles of both hind legs, the neck muscles, diaphragm, intercostal muscle, liver, lung, kidney and oesophagus. It also prescribes a thorough inspection of the whole carcass and offal if any cysticerci are found. However, the firmness by which this routine meat inspection procedure is implemented varies among meat inspectors and abattoirs in Ethiopia. This review identified that the prevalence differed among the reports with the number of organs and muscles inspected. Indeed, higher prevalences were reported by authors who inspected more muscles and organs [21, 32, 33] as compared to those authors who inspected fewer muscle and organs [20, 71, 81]. These variations in meat inspection procedure may strongly affect the reported prevalence, thus hampering direct comparison of studies using different methodologies. A more harmonized meat inspection is consequently highly recommended to allow a more thorough assessment of zonal BCC prevalence.

Furthermore, in this review most reports were based on routine meat inspection; as a result, the limitations of meat inspection, highlighted recently in a Belgian study determining a sensitivity of 0.76% [9], should also be considered in these studies. This could be due to the fact that the cysticerci in light infections might not be evident on routine inspection of the predilection sites [3, 137]. Also, in early infections, viable cysts are inconspicuous in the red meat due to their translucent nature and pinkish-red colour [138]. It also depends on the technical ability and motivation of the meat inspector, for instance, the 4.8% retrospective prevalence report was much lower than the 19.5% reported from a prospective abattoir survey from the same abattoir [42]. Moreover, the speed of the slaughtering activity, the lighting system in the Ethiopian abattoirs and other factors might have contributed to the variation of prevalence reported in Ethiopia [139]. Thus, prevalence reported so far using routine meat inspection in Ethiopia is highly probable an underestimation of the true prevalence.

Despite the above-mentioned methodological problems, there seems to be a variation in the prevalence of BCC and taeniosis in different regions of the country (Fig. 2a). Different factors could have affected these geographical variations in the country; such as the cultural and religious differences in raw beef consumption, the agroecological condition, sanitation conditions (Fig. 2c) and human/cattle population density (Fig. 2b). For instance, the rural communities around Jimma, Borena, Arsi, Bale and south-eastern and north-eastern parts of the country rarely consume raw meat due to cultural and religious reasons [42, 52], which could lead to a lower number of tapeworm carriers potentially contaminating herds and grazing land with *T. saginata* eggs. The high prevalence reports seem to concur with the cattle density (Fig. 2b) and highland altitude which is characterized by a more moderate, colder temperature, high moisture and vegetation coverage as compared to lowlands. Low temperatures are known to favour the survival of *T. saginata* eggs in the environment [5]. However, there are still many data gaps with respect to the research coverage and agroecological factors contributing to parasite survival in the environment, thus urging for more research.

The risk factor analysis showed no significant difference between the sex and age of slaughtered animals with the prevalence of BCC, which is also reported elsewhere [140, 141]. However, reports from some EU countries show significant associations of BCC with age [137], with higher odds in female, older cows [142] and a significant age-sex interaction [143]. Breed and body condition of slaughtered animals were not significantly associated with the odds of BCC, although here again, most of the animals slaughtered were local/zebu and of good body condition. According to this review, the environmental and human characteristics are more responsible for the prevalence variation of BCC in Ethiopia than the animal-related factors.

According to the WHO [3, 144], human taeniosis can be detected by a well-structured questionnaire using the finding of proglottids in stool and underwear as a diagnostic sign. Moreover, as pork is rarely consumed in Ethiopia due to religious and cultural reasons, *T. solium* is less likely to occur, and the detected proglottids are assumed to be *T. saginata.* In this review, a higher prevalence was found using questionnaire-based taeniosis detection than *via* coprological techniques. Coprological techniques have fairly low sensitivities, related to the intermittent egg excretion and depending on the technique used [3, 15]. Also, the questionnaire-based diagnosis is usually not restricted in time, while the stool examinations represent a one-time point. Moreover, in most of such reports, the study subjects were specific groups of the society such as patients visiting health institutions for other illnesses, who are not representative of the general community. Perhaps due to the reason that it is a self-diagnosed and treatable disease, taeniosis patients in Ethiopia visit the hospital less frequently. On the other hand, questionnaire-based studies could also give false positives due to the difficulty to differentiate between other gastrointestinal worm infections and small *T. saginata* proglottids.

Human taeniosis was found strongly associated particularly with raw beef consumption, adults, men and people working in the abattoir and butcher houses. In Ethiopia, mostly adults and particularly males visit restaurants and butchers for beef consumption, often consumed raw. The high prevalence of taeniosis in African countries, some Asian countries, Thailand and Cuba is attributed to the habit of raw or undercooked beef consumption. For instance, the following raw dishes are a potential source of taeniosis: ‘Kitfo’/ ‘Lebileb’ (finely minced beef) and ‘Kurt’ (cubes of beef) in Ethiopia [32], roasted beef over an open fire in Central and East Africa, semi-raw beef dish known as counters “basterma” in Egypt, Turkey and the Middle East [145], tartar shashlik in Russia [146], shish kebab in India [147], larb in Thailand [148] and raw meat in Cuba [149]. Moreover, the average zonal prevalence of BCC or taeniosis at Sidama, Gedeo, or Gurage zone and taenicidal doses/cost at Hawasa and Welayta zone of SNNP region was high, which might be related to the deep-rooted raw beef consumption habit in these communities.

Generally, the higher prevalence of BCC and taeniosis in Ethiopia as opposed to industrialised countries is due to a number of factors such as the deep-rooted raw beef consumption habit mentioned above, but also backyard slaughter lacking meat inspection, poor sanitary infrastructure (Fig. 2c) and improper disposal of sewage. Eggs of *T. saginata* can remain infective for up to 9 months in the soil [150], pasture or water [151]. In Ethiopia, improper application of sewage effluent from sewage treatment plants, or even direct disposal of sewage sludge on the fields could be risky for cattle grazing on contaminated pasture/water. The animal husbandry practice in Ethiopia is mostly extensive. Taeniosis patients in Ethiopia are not regularly treated and the lack of feedback from the slaughterhouse to the farm after detection of a case so that the people on the farm can be tested and treated might also contribute. According to the joint monitoring programme report (UNICEF/WHO 2015) [152], Ethiopia has reduced the proportion of rural population practicing open defecation from 92% in 2000 to 39.1% by 2016. The differences and improvement of the sanitary situation in the rural communities in the recent years might have contributed to the variation in prevalence of changes over time, but none of the papers studied BCC/taeniosis over a longer time period to assess this. Recent reports indicate that around 37% of the total population (over 35 million people) or 43% of the rural population still do not have access to any form of latrine and therefore defecate in the open [28], implying persistence of pasture contamination with taeniid eggs in places where infected people are living.

In Ethiopia, a number of medicinal plants were documented to have taenicidal effect and are still in use by some of the Ethiopian communities where modern health coverage is low or as an alternative to the modern treatment in urban areas. However, the evaluation of the actual efficacy of these plants based on scientifically sound methodologies is not reported.

The economic impact of human taeniosis caused by *T. saginata* is due to treatment costs and/or the number of sick days, and long-term effects. Recently Jansen et al. [8] reported a maximum estimated loss of €795,858 per year from 10,991 taeniosis patients in Belgium, which is related to medication cost, cost of diagnosis and consultation. Since self-medication for gastro-intestinal tract diseases is a common practice in Ethiopia [153], these costs per person might be lower compared to the Belgian estimate. On the other hand, earlier reports from Africa showed a total loss from BCC in Botswana to be near £0.5 million per year while in Kenya it was £1 million [154]. In Iran the direct economic loss from BCC was estimated to be US$ 112,302 [155]. Jansen et al. [8] partitioned the loss incurred to BCC as due to value loss, cost of the inspection, cost of destruction and insurance cost on Belgian cattle. In developing countries like Ethiopia, farmers do not have access to an insurance for BCC, and as such do not have this cost. On the other hand, farmers/butchers do incur the value loss or total loss of the carcass in case of infection. Given the high prevalence of the parasite combined with a large human and cattle population, high economic losses in Ethiopia can be anticipated, though detailed up-to-date investigations on the economic impact of the parasite are currently lacking.

## Conclusions

Prevalence reports of BCC based on routine meat inspection represent an underestimation of the actual prevalence of the parasite in the country. Although the true prevalence of *T. saginata* infection cannot be confirmed based on the current literature, the existing reports are indicative of high prevalence and widespread occurrence in the country. Taeniosis was strongly associated with raw or undercooked beef consumption, occupation, adults and males. An all-inclusive approach to break the life-cycle of the parasite through improving sanitary conditions and strengthening the meat inspection through a harmonized detection and reporting system among abattoirs is suggested. Besides, more research on the risk factors for such a high prevalence of the parasite and detailed investigation on the associated financial losses is needed.

## Supplementary information

**Additional file 1: Table S1.** PRISMA checklist.

**Additional file 2: Table S2.** Prevalence data for bovine cysticercosis in Ethiopia extracted from included articles. **Table S3.** Extracted data for the distribution of cysticerci in organs and carcasses in infected cattle. **Table S4**. Extracted data for cyst viability and average viability proportion calculated. **Table S5**. Data extracted for the prevalence of human taeniosis from the questionnaire-based reports. **Table S6.** Average zonal prevalence of BCC, questionnaire-based and stool test-based taeniosis. **Table S7**. Taenicidal dose and cost based on pharmacy inventory records. **Table S8**. Condemnation of organs and carcasses due to BCC. **Text S1.** References.

**Additional file 3: Figure S1.** Overview of studies reporting human taeniosis (questionnaire-based diagnosis) in Ethiopia. The forest plot contains a horizontal line representing the results of each study and the length of the straight line indicates the 95% CI, the box size the weight of the study and the middle of the box the point estimate of the study. A vertical broken line is the pooled estimate and a diamond shaped box at the bottom is the CI, while the solid line shows the point of null assumption.

## Data Availability

All datasets supporting the conclusions of this article are included within the article and its additional files.

## Abbreviations

Ag-ELISAs: Antigen-enzyme-linked immunosorbent assay
BCC: Bovine cysticercosis
CI: Confidence interval
I^2^: Inverse variance index
IHAT: Indirect hemagglutination test
pOR: Pooled odds ratio
PCR: Polymerase chain reaction
Q: Cochran’s Q test value
OIE: World Animal Health Organisation
RFLP: Restricted fragment length polymorphism
SNNP: Southern Nations and Nationalities and Peoples Region
US$: United States dollar
WHO: World Health Organisation
