# Supplementary material for: Identification of risk areas and practices for Taenia saginata taeniosis/cysticercosis in Ethiopia: a systematic review and meta-analysis
Source: Parasit Vectors. 2020 Jul 29;13:375. doi: 10.1186/s13071-020-04222-y (PMC7391523; doi:10.1186/s13071-020-04222-y)
Supplement: Supplementary file 2 — Additional file 2: Table S2. Prevalence data for bovine cysticercosis in Ethiopia extracted from included articles. Table S3. Extracted data for the distribution of cysticerci in organs and carcasses in infected cattle. Table S4. Extracted data for cyst viability and average viability proportion calculated. Table S5. Data extracted for the prevalence of human taeniosis from the questionnaire-based reports. Table S6. Average zonal prevalence of BCC, questionnaire-based and stool test-based taeniosis. Table S7. Taenicidal dose and cost based on pharmacy inventory records. Table S8. Condemnation of organs and carcasses due to BCC. Text S1. References. [file 13071_2020_4222_MOESM2_ESM.docx]

**Additional file 2**

**Table S2.** Prevalence data for bovine cysticercosis in Ethiopia extracted from included articles

| **Study area/ Abattoir** | **Region** | **Zone** | **Sample size** | **No. positive** | **Prevalence** | **Reference** |
| --- | --- | --- | --- | --- | --- | --- |
| Mekele | Tigray | C. Tigray | 1023 | 74 | 7.2 | [1] |
| Hawasa | SNNP | Sidama | 400 | 105 | 26.2 | [2] |
| Yirgalem | SNNP | Gedeo | 400 | 48 | 12 | [3] |
| Gondar | Amhara | Gondar | 450 | 9 | 2 | [4] |
| Bahir Dar***** | Amhara | W.Gojam | 486 | 94 | 19.4 | [5] |
| Kelem Welega | Oromia | W. Welega | 384 | 25 | 6.5 | [6] |
| Zeway | Oromia | E.Shoa | 400 | 12 | 3 | [7] |
| Ambo | Oromia | W. Shoa | 600 | 93 | 15.5 | [8] |
| Hawasa | SNNP | Sidama | 384 | 88 | 22.9 | [9] |
| Shire | Tigray | N.W. Tigray | 439 | 23 | 5.2 | [10] |
| Debre Zeit | Oromia | E.Shoa | 2022 | 280 | 13.8 | [11] |
| Yabello | Oromia | Borena | 384 | 33 | 8.6 | [12] |
| Babile | Somali | Jigjiga | 384 | 119 | 31 | [13] |
| Dire Dawa | Dire Dawa | Dire Dawa | 384 | 69 | 18.0 | [13] |
| Jigjiga | Somali | Jigjiga | 340 | 114 | 33.5 | [13] |
| Adama | Oromia | E.Shoa | 422 | 83 | 19.7 | [14] |
| Jijiga | Somali | Jijiga | 400 | 9 | 2.2 | [15] |
| Gondar | Amhara | Gondar | 400 | 22 | 5.5 | [16] |
| Debre Zeit | Oromia | E.Shoa | 1292 | 253 | 13.6 | [17] |
| Dukem | Oromia | E.Shoa |  |  | 19.2 | [17] |
| Modjo | Oromia | E.Shoa |  |  | 17.9 | [17] |
| Modjo Luna | Oromia | E.Shoa |  |  | 27.6 | [17] |
| Gondar | Amhara | Gondar | 1168 | 113 | 9.7 | [18] |
| Addis Ababa | AA | AA | 44000 | 815 | 1.9 | [19] |
| Modjo Luna | Oromia | E.Shoa | 17000 | 1450 | 8.5 | [19] |
| Asella | Oromia | Arsi | 430 | 5 | 1.2 | [20] |
| Asella | Oromia | Arsi | 450 | 22 | 4.9 | [21] |
| Nekemte | Oromia | E. Welega | 410 | 11 | 2.7 | [22] |
| Bishoftu | Oromia | E.Shoa | 430 | 24 | 5.6 | [23] |
| Kombolcha | Amhara | S. Wollo | 420 | 28 | 6.7 | [24] |
| Jimma | Oromia | Jimma | 547 | 28 | 5.12 | [25] |
| Shire | Tigray | N.W. Tigray | 400 | 66 | 15.6 | [26] |
| Mekele | Tigray | C. Tigray | 1800 | 80 | 4.44 | [27] |
| Halab Kulito | SNNP | Halaba | 384 | 33 | 8.6 | [28] |
| Addis Ababa | AA | AA | 535 | 19 | 3.5 | [29] |
| Nekemte | Oromia | E. Welega | 1355 | 286 | 21 | [30] |
| Bedele | Oromia | Buno Bedele | 550 | 11 | 2.0 | [31] |
| Kombolcha | Amhara | S. Wollo | 425 | 20 | 4.7 | [32] |
| Bahir Dar | Amhara | W.Gojam | 918 | 193 | 21.0 | [33] |
| Addis Ababa | Addis Ababa | AA | 522 | 39 | 7.5 | [34] |
| Addis Ababa | Addis Ababa | AA | 11227 | 842 | 7.5 | [35] |
| Dangila | Amhara | Awi | 480 | 85 | 17.7 | [33] |
| Debre Markos | Amhara | E.Gojam | 668 | 74 | 11.1 | [33] |
| Dejen | Amhara | E.Gojam | 401 | 74 | 18.4 | [33] |
| Finote Selam | Amhara | W.Gojam | 452 | 81 | 17.9 | [33] |
| Gondar | Amhara | Gondar | 870 | 157 | 18.1 | [33] |
| Injibara | Amhara | Awi | 361 | 78 | 21.6 | [33] |
| Meraw | Amhara | W.Gojam | 306 | 82 | 26.8 | [33] |
| Gondar | Amhara | Gondar | 400 | 9 | 2.2 | [36] |
| Part of Tigray | Tigray | C. Tigray | 3711 | 308 | 8.3 | [37] |
| Wolaita | SNNP | Wolaita | 420 | 37 | 8.8 | [38] |
| Jimma | Oromia | Jimma | 500 | 22 | 4.4 | [39] |
| Bahir Dar | Amhara | W.Gojam | 465 | 12 | 2.6 | [40] |
| West Arsi | Oromia | W. Arsi | 768 | 38 | 4.9 | [41] |
| Dire Dawa | Dire Dawa | Dire Dawa | 33298 | 4395 | 13.2 | [42] |
| Shashemene | Oromia | W. Arsi | 405 | 41 | 10.12 | [43] |
| Gondar | Amhara | Gondar | 768 | 23 | 2.9 | [44] |
| Wolaita Sodo | SNNP | Wolaita | 415 | 47 | 11.3 | [45] |
| Woliso | Oromia | S.W. Shoa | 830 | 40 | 4.8 | [46] |
| Jimma | Oromia | Jimma | 600 | 15 | 2.5 | [47] |
| Bahir Dar | Amhara | W.Gojam | 480 | 20 | 4.2 | [48] |
| Jimma | Oromia | Jimma | 520 | 19 | 3.6 | [49] |
| Kombolcha | Amhara | S. Wollo | 234 | 21 | 8.9 | [50] |
| Batu | Oromia | E.Shoa | 384 | 10 | 2.6 | [51] |
| Harar | Hareri | Harar | 898 | 177 | 19.7 | [52] |
| Adgrat (E.Tigray) | Tigray | E. Tigray | 540 | 29 | 5.4 | [53] |
| Wolaita | SNNP | Wolaita | 540 | 14 | 2.6 | [54] |
| Debre Zeit | Oromia | E.Shoa | 700 | 38 | 5.4 | [55] |
| Jimma | Oromia | Jimma | 512 | 15 | 2.9 | [56] |
| Adama | Oromia | E.Shoa | 384 | 10 | 2.6 | [57] |
| Debre Berhan | Amhara | North Shoa | 384 | 18 | 6.6 | [58] |
| W. Shoa | Oromia | W. Shoa | 600 | 15 | 2.5 | [59] |
| Desie | Amhara | S.Wollo | 384 | 26 | 6.8 | [60] |
| Debre Berhan | Amhara | North Shoa | 405 | 22 | 5.4 | [61] |
| Central & EE* | AA& EE | AA & EE | 41 | 38 | 92.7 | [62] |
| Addis Ababa** | AA | AA | 743 | 190 | 25.7 | [34] |

****** Serological study IHAT, * molecular study, W.-West, E.- East, N.-North, S.-South C.-Central, AA-Addis Ababa, and EE- Eastern Ethiopia

**Table S3.** Extracted data for distribution of cysticerci in organs and carcasses in infected cattle.

| Study area | Region | Sample size | Prevalence | Shoulder | Heart | Tongue | Masseter | Liver | Thigh M | Dg | Lung | Kidney | ICM | NM | IM | Spleen | LDM | Reference |
| --- | --- | --- | --- | --- | --- | --- | --- | --- | --- | --- | --- | --- | --- | --- | --- | --- | --- | --- |
| Mekele | Tigray | 1023 | 7.2 | 3 | 4 | 4 | 1 | 65 |  | 0 | 3 |  |  |  |  |  |  | [1] |
| Hawasa | SNNP | 400 | 26.3 | 36 | 45 | 13 | 34 | 3 | 0 | 7 | 2 | 1 | 0 |  |  |  |  | [2] |
| Yirgalem | SNNP | 400 | 12.0 | 10 | 13 | 45 | 26 | 16 |  |  | 0 |  |  |  |  |  |  | [3] |
| Gondar | Amhara | 450 | 2.0 | 5 | 0 | 1 | 3 |  |  | 0 |  |  |  |  |  |  |  | [4] |
| Kelem Welega | Oromia | 383 | 6.5 | 13 | 2 | 3 | 6 | 1 |  |  | 0 | 0 |  |  |  | 0 |  | [6] |
| Zeway | Oromia | 400 | 3.0 | 21 | 27 | 33 | 15 |  |  |  |  |  |  |  |  |  |  | [7] |
| Ambo | Oromia | 600 | 15.5 | 23 | 10 | 9 | 13 | 4 | 17 | 1 |  |  | 2 |  |  |  |  | [8] |
| Hawasa | SNNP | 384 | 22.9 | 25 | 20 | 31 | 12 | 0 |  |  | 0 |  |  |  |  |  |  | [9] |
| Shire | Tigray | 439 | 5.2 | 0 | 2 | 2 | 0 | 19 |  |  |  |  |  |  |  |  |  | [10] |
| Yabello | Oromia | 384 | 8.6 | 28 | 14 | 12 | 28 | 7 |  | 13 |  |  |  |  |  |  |  | [12] |
| Dire Dawa | Dire Dawa | 384 | 18.0 | 66 | 26 | 94 | 56 | 106 | 20 | 4 |  |  | 0 |  |  |  |  | [13] |
| Adama | Oromia | 422 | 19.7 |  | 121 | 158 | 77 | 0 |  |  | 0 | 7 |  |  |  | 0 |  | [14] |
| Jijiga | Jigjiga | 400 | 2.3 | 6 | 7 | 9 | 3 | 1 |  |  |  |  |  |  |  |  |  | [15] |
| Gondar | Amhara | 400 | 5.5 | 9 | 14 | 11 | 10 |  |  |  |  |  |  |  |  |  |  | [16] |
| E. Shoa | Oromia | 1292 | 19.6 | 161 | 191 | 197 | 90 | 20 |  |  | 10 |  |  |  |  |  |  | [17] |
| AA | AA | 61000 | 3.7 | 326 | 691 | 551 |  | 95 |  |  |  |  |  |  |  |  |  | [19] |
| Asella | Oromia | 430 | 1.2 |  | 5 | 2 | 2 | 7 |  |  | 0 |  |  |  |  |  |  | [20] |
| Nekemte | Oromia | 410 | 2.7 | 5 | 0 | 5 | 3 |  |  | 0 |  |  |  |  |  |  |  | [22] |
| Bishoftu | Oromia | 430 | 5.6 | 15 | 5 | 22 | 11 |  |  | 1 |  |  |  |  |  |  |  | [23] |
| Kombolcha | Amhara | 420 | 6.7 | 67 | 33 | 27 | 23 | 6 |  | 4 |  |  |  |  |  |  |  | [24] |
| Jimma | Oromia | 547 | 5.1 | 16 | 1 |  |  | 1 | 2 | 1 |  |  |  |  |  |  | 13 | [25] |
| Shire | Tigray | 400 | 16.5 | 20 | 9 | 10 | 11 | 5 | 18 | 1 |  |  | 3 |  |  | 0 |  | [26] |
| Mekele | Tigray | 1800 | 4.4 |  | 4 | 7 | 3 | 55 |  |  | 12 |  |  |  |  |  |  | [27] |
| Halaba | SNNP | 384 | 8.6 | 0 | 26 | 26 | 24 |  |  | 30 |  |  | 28 |  |  |  |  | [28] |
| AA | AA | 535 | 3.6 | 10 | 2 | 5 | 4 | 1 |  | 2 |  |  |  |  |  |  |  | [29] |
| Netemte | Oromia | 1355 | 21.1 | 221 | 255 | 180 | 144 | 15 | 103 | 71 |  |  | 32 | 71 |  |  | 57 | [30] |
| Bedele | Oromia | 550 | 2.0 | 3 | 4 | 4 |  |  |  |  |  |  |  |  |  |  |  | [31] |
| Kombolcha | Amhara | 425 | 4.7 |  | 12 | 7 | 5 | 2 |  | 1 |  |  |  |  |  |  |  | [32] |
| AA | AA | 11227 | 7.5 | 281 | 294 | 300 | 308 | 23 | 101 | 94 |  |  | 12 |  |  |  |  | [35] |
| N.W. Ethiopia | Amhara | 4456 | 18.5 | 211 | 212 | 318 | 301 | 13 | 181 | 86 | 0 | 0 | 4 |  | 1 | 8 |  | [33] |
| Gondar | Amhara | 400 | 2.3 | 4 | 1 | 2 | 2 |  |  |  |  |  | 0 |  |  |  |  | [36] |
| Part of Tigray | Tigray | 3711 | 8.3 | 10 | 10 | 23 | 22 | 272 |  |  |  |  |  |  |  |  |  | [37] |
| Wolaita | SNNP | 420 | 8.8 | 18 |  | 13 | 9 | 0 |  |  |  |  |  |  |  |  |  | [38] |
| Jimma | Oromia | 500 | 4.4 | 11 | 4 | 20 | 4 | 0 |  |  | 0 | 0 |  |  |  | 0 |  | [39] |
| Bahir Dar | Amhara | 465 | 2.6 |  | 6 | 10 | 9 | 1 |  |  |  |  |  |  |  |  |  | [40] |
| W.Arsi | Oromia | 768 | 4.9 |  | 13 | 8 | 8 | 6 | 3 |  |  |  |  |  |  |  |  | [41] |
| Shashemene | Oromia | 405 | 1.7 |  | 30 | 32 | 15 |  |  |  |  | 1 |  |  |  | 0 |  | [43] |
| Gondar | Amhara | 768 | 3.0 | 11 | 15 | 6 | 10 | 1 |  |  |  |  |  |  |  |  |  | [44] |
| Wolaita | SNNP | 415 | 11.3 | 21 | 29 | 15 | 17 | 0 |  |  | 0 |  |  |  |  |  |  | [45] |
| S.W.Shoa | Oromia | 1216 | 4.6 |  | 23 | 17 | 8 | 2 |  | 6 |  |  |  |  |  |  |  | [46] |
| Jimma | Oromia | 600 | 2.5 | 12 | 5 | 5 | 4 | 4 |  |  |  | 2 |  | 9 |  |  |  | [47] |
| Bahir Dar | Amhara | 480 | 4.2 | 10 | 4 | 12 | 7 | 1 |  |  |  |  |  |  |  |  |  | [48] |
| Jimma | Oromia | 520 | 3.7 | 17 | 8 | 8 | 4 | 4 |  | 2 |  | 3 |  | 11 |  |  |  | [49] |
| Kombolcha | Amhara | 234 | 9.0 | 10 | 11 | 9 | 11 | 13 |  | 5 | 2 |  |  |  |  |  |  | [50] |
| Batu | Oromia | 384 | 2.6 | 2 | 4 | 5 | 4 |  |  |  |  |  |  |  |  |  |  | [51] |
| Harar | Hareri | 898 | 19.7 | 74 | 30 | 45 | 39 | 55 |  |  | 0 |  |  |  |  |  |  | [52] |
| Eastern Tigray | Tigray | 540 | 5.4 | 9 | 19 | 14 | 10 | 7 | 5 |  | 0 |  |  |  |  |  |  | [53] |
| Wolaita | SNNP | 540 | 2.6 | 5 | 6 | 12 | 9 |  |  | 2 |  |  |  |  |  |  |  | [54] |
| Debre Zeit | Oromia | 700 | 5.4 | 21 | 10 | 6 | 4 |  |  |  |  |  |  |  |  |  |  | [55] |
| Adama | Oromia | 383 | 2.6 | 4 | 2 | 12 | 3 | 4 |  |  |  |  |  |  |  |  |  | [57] |
| Debre Brehan | Amhara | 384 | 4.7 | 18 | 12 | 15 | 17 |  |  | 8 |  |  | 5 |  |  |  |  | [58] |
| West Shewa | Oromia | 600 | 2.5 | 15 | 3 | 7 | 5 |  |  |  |  |  |  |  |  |  |  | [59] |
| Dessie | Amhara | 384 | 6.8 | 8 | 2 | 13 | 6 | 1 |  |  |  | 0 |  |  |  |  |  | [60] |
| Debre Brehan | Amhara | 405 | 5.4 | 4 | 6 | 7 | 4 | 1 |  |  |  |  |  |  | 1 |  |  | [61] |
|  | **Total** | **108250** |  | 1865 | 2302 | 2402 | 1444 | 837 | 450 | 339 | 29 | 14 | 82 | 91 | 2 | 8 | 70 |  |
| **Total number of infected organs and muscles** | | | | | | | **9935** |  |  |  |  |  |  |  |  |  |  |  |
|  | **Percent positive** | | | 18.77 | 23.17 | 24.18 | 14.53 | 8.42 | 4.53 | 3.41 | 0.29 | 0.14 | 0.83 | 0.92 | 0.02 | 0.08 | 0.70 |  |

IC- intercostal muscle, No- Number, +ve -Positive, IM -intestinal mucosa, ThM- thigh muscle, Dg- Diaphragm, NM -neck muscle, LDM- Longissimus dorsi muscle, the numbers under the listed organs represents number of positive organs from the infected cattle

**Table S4**. Extracted data for cyst viability and average viability proportion calculated

| Study A | Sample size | No. of BCC positive % | No. of cysts collected | No. of viable cyst | Percent of viable cyst | Reference |
| --- | --- | --- | --- | --- | --- | --- |
| Hawasa | 400 | 105 (26.3) | 500 | 221 | 44.2 | [2] |
| Yirgalem | 400 | 48 (12.0) | 190 | 89 | 46.8 | [3] |
| Zeway | 400 | 12 (3.0) | 96 | 32 | 33.3 | [7] |
| Ambo | 600 | 93 (15.5) | 122 | 95 | 77.9 | [8] |
| Hawasa | 384 | 88 (22.9) | 88 | 55 | 62.5 | [9] |
| Yabello | 384 | 33 (8.6) | 102 | 31 | 30.4 | [12] |
| Adama | 422 | 83 (19.7) | 192 | 82 | 42.7 | [14] |
| Jijiga | 400 | 9 (2.3) | 24 | 8 | 33.3 | [15] |
| Gondar | 400 | 22 (5.5) | 44 | 14 | 31.8 | [16] |
| East Shoa | 1292 | 253 (19.6) | 478 | 305 | 63.8 | [17] |
| Asella | 430 | 5 (1.2) | 16 | 6 | 37.5 | [20] |
| Bishoftu | 430 | 24 (5.6) | 54 | 36 | 66.7 | [23] |
| Kombolcha | 420 | 28 (6.7) | 160 | 110 | 68.8 | [24] |
| Jimma | 547 | 28 (5.1) | 102 | 45 | 44.1 | [25] |
| Shire Indesillse | 400 | 66 (15.6) | 120 | 35 | 29.2 | [26] |
| Addis Ababa | 535 | 19 (3.6) | 24 | 11 | 45.8 | [29] |
| Kombolcha | 425 | 20 (4.7) | 49 | 27 | 55.1 | [32] |
| N.W. Ethiopia | 4456 | 824 (18.5) | 824 | 705 | 85.6 | [33] |
| Addis Ababa | 11227 | 842 (7.5) | 842 | 704 | 83.6 | [35] |
| Gondar | 400 | 9 (2.3) | 29 | 11 | 37.9 | [36] |
| Wolaita | 420 | 37 (8.8) | 91 | 62 | 68.1 | [38] |
| Jimma | 500 | 22 (4.4) | 114 | 49 | 43.0 | [39] |
| Bahir Dar | 465 | 12 (2.6) | 56 | 19 | 33.9 | [40] |
| Shashemene | 405 | 41 (10.1) | 178 | 60 | 33.7 | [43] |
| Gondar | 768 | 23 (2.9) | 32 | 11 | 34.4 | [44] |
| Wolaita Sodo | 415 | 47 (11.3) | 304 | 86 | 28.3 | [45] |
| S.W. Shoa | 1216 | 56 (4.6) | 56 | 22 | 39.3 | [46] |
| Bahir Dar | 480 | 20 (4.2) | 119 | 73 | 61.3 | [48] |
| Jimma | 520 | 19 (3.6) | 57 | 15 | 26.3 | [49] |
| Batu | 384 | 10 (2.6) | 24 | 14 | 58.3 | [51] |
| Harar | 898 | 177 (19.7) | 661 | 185 | 28.0 | [52] |
| Debre Zeit | 700 | 38 (5.4) | 126 | 64 | 50.8 | [55] |
| Wolaita | 540 | 15 (2.8) | 124 | 49 | 39.5 | [54] |
| Jimma | 512 | 15 (2.9) | 109 | 47 | 43.1 | [56] |
| Adama | 384 | 10 (2.6) | 65 | 43 | 66.2 | [57] |
| W. Shewa | 600 | 15 (2.5) | 15 | 7 | 46.7 | [59] |
| **Total** | **34159** |  | **6309** | **3523** | 55.8 |  |

**Table S5**. Data extracted for the prevalence of human taeniosis from the questionnaire-based reports

| **Study region** | **Region** | **Zone** | **Sample size** | **No. positive** | **Percent** | **Infection period** | **Reference** |
| --- | --- | --- | --- | --- | --- | --- | --- |
| Hawasa | SNNP | Sidama | 120 | 77 | 64.2 | Not indicated | [2] |
| Yirgalem | SNNP | Gedio | 170 | 119 | 70 | In the course of life | [3] |
| Zeway | Oromia | E. Shoa | 120 | 68 | 56.7 | At least once in life | [7] |
| Ambo | Oromia | W. Shoa | 180 | 105 | 58.3 | At least once in life | [8] |
| Hawasa | SNNP | Sidama | 50 | 22 | 44 | Not indicated | [9] |
| Dire Dawa | Dire Dawa | Dire Dawa | 300 | 133 | 44.3 | Different time | [13] |
| Jigjiga | Somali | Jigjiga | 600 | 299 | 49.8 | Different time | [13] |
| E. Shoa | Oromia | East Shoa | 396 | 315 | 79.5 | At least once in life | [17] |
| Asella | Oromia | Arsi | 415 | 184 | 44.3 | At least once in life | [20] |
| Asella | Oromia | Arsi | 125 | 89 | 71.2 | At least once in life | [21] |
| Nekemte | Oromia | E.Welega | 103 | 14 | 13.5 | Had contracted | [22] |
| Bishoftu | Oromia | E. Shoa | 100 | 64 | 64 | Not indicated | [23] |
| Kombolcha | Amhara | South Wollo | 200 | 62 | 31 | At least once in life | [24] |
| Jimma | Oromia | Jimma | 86 | 41 | 47.6 | At least once in life | [25] |
| Shire | Tigray | N. W. Tigray | 270 | 120 | 44.4 | At least once in life | [26] |
| Kulito | SNNP | Halaba SP | 100 | 19 | 19 | At least once in life | [28] |
| Bedele | Oromia | Buno Bedele | 52 | 23 | 44.2 | Not indicated | [31] |
| Jimma | Oromia | Jimma | 60 | 34 | 56.7 | At least once in life | [39] |
| Shashemene | Oromia | W. Arsi | 61 | 28 | 45.9 | At least once in life | [43] |
| Wolaita | SNNP | Wolayita | 79 | 40 | 50.6 | At least once in life | [45] |
| S.W. Shoa | Oromia | S. W. Shoa | 392 | 216 | 55.1 | At least once in life | [46] |
| Jimma | Oromia | Jimma | 120 | 80 | 66.7 | At least once in life | [47] |
| Bahir Dar | Amhara | West Gojam | 69 | 21 | 30.4 | Had contracted | [48] |
| Jimma | Oromia | Jimma | 180 | 116 | 64.4 | In the last two years | [49] |
| Kombolcha | Amhara | South Wollo | 110 | 40 | 36.6 | Not indicated | [50] |
| Batu | Oromia | E. Shoa | 100 | 59 | 59 | At least once in life | [51] |
| Harar | Hareri | Hareri | 300 | 182 | 60.7 | In the last 2 years | [52] |
| Wolaita | SNNP | Wolayita | 80 | 50 | 62.5 | At least once in life | [54] |
| Bishoftu | Oromia | East Shoa | 200 | 139 | 69.5 | At least once in life | [55] |
| Adama | Oromia | E. Shoa | 200 | 91 | 45.5 | At least once in life | [57] |
| Debre Berhan | Amhara | North Shoa | 80 | 22 | 27.5 | At least once in life | [58] |
| West Shoa | Oromia | W. Shoa | 110 | 70 | 63.6 | Not specified | [59] |
| Jimma | Oromia | Jimma | 121 | 100 | 82.6 | At least once in life | [63] |

**Table S6.** Average zonal prevalence of BCC, questionnaire based and stool test based taeniosis

| Region | Zone | Average BCC prevalence | Average QB taeniosis prevalence | Average zonal STB taeniosis prevalence |
| --- | --- | --- | --- | --- |
| Addis Ababa | Addis Ababa | 3.05 | No data | 3.69 |
| Amhara | Awi | 19.66 | No data | No data |
| Amhara | East Gojam | 13.84 | No data | 1.04 |
| Amhara | North Gonder | 8.21 | No data | 0.62 |
| Amhara | North Shewa | 5.07 | 27.5 | 3.79 |
| Amhara | South Wollo | 6.49 | 32.9 | 1.29 |
| Amhara | West Gojam | 15.51 | 30.4 | 1.16 |
| Dire Dawa | Dire Dawa | 13.25 | 44.3 | No data |
| Hareri | Hareri | 19.71 | 60.7 | No data |
| Oromia | Arsi | 3.07 | 55.5 | No data |
| Oromia | Bale | No data | No data | 1.33 |
| Oromia | Borena | 8.6 | No data | No data |
| Oromia | East Harerge | No data | No data | 1.2 |
| Oromia | East Shewa | 11.77 | 65.95 | 3.95 |
| Oromia | East Wellega | 16.83 | 42.04 | 1.34 |
| Oromia | Ilubabor/Bedele | 2 | 45.09 | No data |
| Oromia | Jimma | 3.69 | 65.43 | 1.81 |
| Oromia | North Shewa | No data | No data | 2.65 |
| Oromia | South West Shewa | 4.82 | 63.6 | No data |
| Oromia | West Arsi | 6.73 | 45.9 | No data |
| Oromia | West Shewa | 9 | 60.34 | No data |
| Oromia | West Wellega | 6.5 | No data | No data |
| SNNPR | Alaba | 8.6 | 19 | No data |
| SNNPR | Gamo Gofa | No data | No data | 1.06 |
| SNNPR | Gedio | 12 | 70 | 1.31 |
| SNNPR | Gurage | No data | No data | 10.73 |
| SNNPR | Sidama | 24.62 | 58.24 | 2.64 |
| SNNPR | Wolayita | 7.2 | 56.6 | 4.38 |
| Somali | Fafan | 21.5 | 49.8 | 1.2 |
| Tigray | Central | 7.07 | No data | 0.57 |
| Tigray | Eastern | 5.37 | No data | No data |
| Tigray | Western | 10.61 | 44.4 | 1.46 |

QB, questionnaire based, STB, stool test based

**Table S7**. Taenicidal dose and cost based on pharmacy inventory records

| Region/Town | Data period | No. Pharmacy | Dose/years | Worth in ETB/USD | Reference |
| --- | --- | --- | --- | --- | --- |
| Hawasa | 2005-2009 | 8 | 472,013/5yrs | 1,416,039/88,500 | [3] |
| Ziway | 2009-2010 | 6 | 74,614/2yrs. | 110,560/20,729 | [7] |
| Hawasa | 2008-2009 | Not specified | 92,203/2yrs. | 184,406/5,763 | [9] |
| Gonder | 2013-2014 | Not specified | 50,969/2yrs. | 193,558/10,048 | [16] |
| Jimma | 2013-2014 | 10 | 22, 936/yr. | 67,950/3,527 | [25] |
| Halaba | 2014-2015 | Not specified | 63461/2year | 190362/ 9285.9 | [28] |
| Jimma | 2007-2008 | 8 | 103,596/2yrs | 222,706/22,270 | [39] |
| Shashemene | 2009-2011 | Not specified | 61028/3rys. | 192,071/12,004 | [43] |
| Wolaita | 2004-2007 | 7 | 335,772/4yrs | 886,447/93,310 | [45] |
| Kombolcha | 2016 | Not specified | 6,998/year | 19,621.00/… | [50] |
| Wolaita | 2009-2010 | Not specified | 29,952/yr. | 40,202/2,407 | [54] |
| Debre Zeit | 2010 | 6 | 29,272/yr. | 114,293/7,143 | [55] |
| Adama | 2011-2013 | Not specified | Not specified/3yrs | 378,610/18,379 | [57] |
| W. Shoa | 2013 | 7 | 68,181/yr. | 380,032/19,729 | [59] |

**Table S8**. Condemnation of organs and carcasses due to BCC

| **Organ/**  **Carcass** | **Condemnation status** | **Reference** | | | | | | | | | | | | | | **Total** | **% due to BCC** | |
| --- | --- | --- | --- | --- | --- | --- | --- | --- | --- | --- | --- | --- | --- | --- | --- | --- | --- | --- |
|  |  | [64] | [65] | [66] | [67] | [68] | [69] | [70] | [71] | [72] | [73] | [74] | [75] | [76] |  | |  | |
| Liver | Total No. | 131 | 569 | 944 | 228 | 171 | 532 | 180 | 531 | 311 | 49275 | 183 | 21780 | 372 | 75207 | |  | |
|  | By BCC | 19 | 60 |  |  |  |  |  | 64 | 6 | 3425 | 0 | 0 |  | 3574 | | 4.75 | |
| Lung | Total No. | 61 | 368 | 911 | 234 | 137 | 305 | 67 | 302 | 142 | 57507 | 88 | 11561 | 372 | 72055 | |  | |
|  | By BCC |  |  | 3 |  |  |  |  | 1 |  | 0 | 0 | 0 |  | 4 | | 0.01 | |
| Kidney | Total No. | 9 | 252 | 17 | 30 | 25 | 17 | 5 | 59 | 39 | 13315 | 3 |  |  | 13772 | |  | |
|  | By BCC |  | 70 | 3 |  |  |  |  | 1 |  | 0 | 0 |  |  | 74 | | 0.54 | |
| Heart | Total No. | 2 | 264 | 116 | 42 | 26 | 32 |  | 38 | 34 | 20003 | 9 | 4797 | 4 | 25367 | |  | |
|  | By BCC | 2 | 107 | 34 | 24 |  |  |  | 8 |  | 974 | 1 | 2695 |  | 3837 | | 15.13 | |
| Tongue | Total No. |  | 53 |  |  | 9 | 7 | 3 | 9 | NE | 4243 | 3 | 3880 | 5 | 8212 | |  | |
|  | By BCC |  | 27 |  |  | 4 | 3 | 3 | 1 |  | 495 | 3 | 2542 | 5 | 3083 | | 37.54 | |
| Carcass partial | Total No. | NE | 184 | NE | NE | NE | 36 |  | NE | NE | 52437 | 2 | 132 |  | 52791 | |  | |
|  | By BCC |  | 83 |  |  |  | 1 |  |  |  | 8 | 2 | 0 |  | 94 | | 0.18 | |
| Carcass total | Total No. | NE |  | NE | NE | NE |  | 29 | NE | NE | 142 |  | 53 | 2 | 226 | |  | |
|  | By BCC |  |  |  |  |  |  | 1 |  |  | 4 |  | 7 | 1 | 12 | | 5.31 | |
| Head | Total No. | NE |  | NE | NE | NE |  |  | NE | NE | 26396 |  | 3159 |  | 29555 | |  | |
|  | By BCC | N |  |  |  | N |  |  |  |  | 63 |  | 1116 |  | 1179 | | 3.99 | |
| Total No. inspected | | 745 | 1152 | 3675 | 406 | 384 | 1550 | 433 | 1022 | 768 | 62917 | 534 | 33,298 | 600 | 102921 | |  | |
| Total carcasses and organs condemned | | | | | | 277185 | | | | | | | | | | |  |  |
| Total condemnation due to BCC | | | | | | 11857 | | | | | | | | | | | 4.28 |  |

**Text S1. References for Additional file 2**

1. Abay G, Kumar A. Cysticercosis in cattle and its public health implications in Mekelle city and surrounding areas, Ethiopia. Ethiop Vet J. 2013;17:31–40.
2. Abunna F, Tilahun G, Megersa B, Regassa A, Kumsa B. Bovine cysticercosis in cattle slaughtered at Awassa municipal abattoir, Ethiopia: prevalence, cyst viability, distribution and its public health implication. Zoonoses Public Health. 2008;55:82–8.
3. Abunna F. Prevalence, organ distribution, viability and socioeconomic implication of bovine cysticercosis/teniasis, Ethiopia. Rev Elev Med Vet Pays Trop. 2013;66:25–30.
4. Adem E, Alemneh T. The occurrence of *Cysticercus bovis* at Gondar Elfora abattoir, northwest of Ethiopia. J Cell Anim Biol. 2016;10:16-21.
5. Alemu M. Bovine cysticercosis: prevalence, economic and public health importance. DVM, thesis, FVM, Addis Ababa University, Debre Zeit, Ethiopia; 1997.
6. Bayou K, Taddesse T. Prevalence of bovine cysticercosis of slaughtered cattle in Dale Wabera district municipal abattoir, western Ethiopia. SM Vet Med Anim Sci Res. 2018;1:1001.
7. Bedu H, Tafess K, Shelima B, Woldeyohannes D, Amare B, Kassu A. Bovine cysticercosis in cattle slaughtered at Zeway municipal abattoir: prevalence and its public health importance. J Vet Sci Technol. 2011; doi:10.4172/21577579.1000108.
8. Bekele D, Berhanu B, Pal M. Studies on the prevalence, cyst viability, organ distribution and public health significance of bovine cysticercosis in Ambo municipality abattoir, western Shoa, Ethiopia. J Parasitol Vector Biol. 2017;9:73–80.
9. Belachew M, Ibrahim N. Prevalence of *cysticercus bovis* in Hawassa municipal abattoir and its public health implication. Am-Euras J Sci Res. 2012;7:238–45.
10. Belay S, Afera B. Prevalence of *cysticercus bovis* in cattle at municipal abbatoir of Shire. J Vet Sci Technol. 2014; doi:10.4172/2157-7579.1000196.
11. Belayneh G. Prevalence and significance of *cysticercus bovis* among cattle slaughtered at Debre Zeit abattoir, DVM thesis, FVM, Addis Ababa University, Debre Zeit, Ethiopia;1990.
12. Beyene T, Hiko A. Zoonotic metacestodes and associated financial loss from cattle slaughtered at Yabello municipal abattoir, Borana-Oromia, Ethiopia. Parasite Epidemiol Control. 2019;3:e00096.
13. Biza A. Prevalence of bovine cysticercosis and human taeniasis in Jigjiga, Babile and Dire Dawa towns, eastern Ethiopia. MSc Thesis, Haramaya University, Haramaya, Ethiopia; 2018.
14. Birhanu T, Abda S. Prevalence, economic impact and public perception of hydatid cyst and *cysticercus bovis* on cattle slaughtered at Adama municipal abattoir, south eastern Ethiopia. Am-Euras J Sci Res. 2014;9:87–97.
15. Biruk WA. Prevalence of bovine cysticercosis at Jijiga municipal abattoir, Ethiopia. J Vet Sci Technol. 2017; doi:10.4262/2157-7579.1000442.
16. Cheru H, Zerihun T. Prevalence and public health significance of *cysticercus bovis* in cattle slaughtered at Gondar Elfora Abattior. Biomed Nurs. 2017; doi:10.7537/marsbnj030217.09.
17. Degefu H. Prevalence and risk factors for *Taena saginata* taeniasis/ cysticercosis in three selected areas of eastern Shoa. MSc Thesis, Addis Ababa University, Debre Zeit, Ethiopia; 2005.
18. Demisie A. Prevalence and significant of *cycticercus bovis* among cattle slaughtered at Gonder meat factory. DVM thesis, Addis Ababa University, Debre zeit, Ethiopia; 1989.
19. Deressa A, Yohannes M, Alemayehu M, Degefu H, Tolosa T, Pal M. Human taeniasis in health centers and bovine cysticercosis in selected abattoirs in Addis Ababa and Modjo, Ethiopia. Int J Livest Health. 2012; 2:217–26.
20. Edao A, Dima FG, Deressa FB. Prevalence of bovine cysticercosis and status of human taeniasis in and around Asella town, Tiyoworeda, south east Ethiopia. Glob J Med Res. 2016;16:18–26.
21. Elemo KK, Hiko A, Dawud A. Bovine cysticercosis and human taeniasis with public health implication at Asella town, Arsi zone of Oromia regional state, Ethiopia. Bull African Anim Heal Prod. 2017;65:49-59
22. Emiru W, Hiluf G. Study on prevalence of bovine cystcercosis and public health importance of taeniosis at the Nekemte municipal abattoir, Nekemte, western Oromia. J Dairy Vet Sci. 2019; doi:10.19080/JDVS.2019.09.555754.
23. Emiru L, Tadesse D, Kifleyohannes T, Sori T, Hagos Y. Prevalence and public health significance of bovine cysticercosis at Elfora abattoir, Bishoftu, Ethiopia. J Public Health Epidemiol. 2015;7:34–40.
24. Endris J, Nigussie H. Bovine cysticercosis: prevalence, cyst viabilty and distribution in cattle slaughtered at Kombolcha Elfora meat factory, Ethiopia. Am-Euras J Sci Res. 2011;11:173–6.
25. Firew F, Moges N. Prevalence of bovine cysticercosis in cattle and zoonotic significance in Jimma town, Ethiopia. Acta Parasitol Glob. 2014;5:214–22.
26. Gebremichael D, Mohammed T. Risk factors and public health significance of cysticercosis in cattle and human in Shire Indasilassie district, northern Ethiopia. Adv Biol Res. 2013;7:282–7.
27. Getachew T, Olani W, Sadia H. Prevalence and economic significance of bovine hydatidosis and cysticercosis in Mekelle municipality abattoir, northern Ethiopia. J Vet Sci Res. 2017;2: 000135.
28. Hirpha A, Bekele T, Melaku M. Study on bovine cysticercosis with special attention to its prevalence, economic losses and public health significance in and around Halaba Kulito town, south Ethiopia. World J Agric Sci. 2016;12:299–307.
29. Ibrahim N, Zerihun F. Prevalence of *Tania saginata* cysticercosis in cattle slaughtered in Addis Ababa municipal abattoir, Ethiopia. Glob Vet. 2012;8:467–71.
30. Ibrahim A. Bovine cysticercosis in animals slaughtered in Nekemte municipality slaughter house. In: Ethiopian veterinary association proceedings of the 14th conference, Addis Ababa, Ethiopia; 2000. p. 46–55.
31. Kano H, Tulu D. Prevalence and socio-economic importance of major bovine metacestodes in Bedele municipal abattoir, south western Ethiopia. Int J Adv Agric Sci Technol. 2018;5:71–85.
32. Kassaw M, Belay W, Tesfaye W. Prevalence of *cysticercus bovis* in cattle slaughterd at Kombolcha Elfora meat processing factory, northern Ethiopia. Int J Curr Res Biol Med. 2017;2:1–6.
33. Kebede N. Cysticercosis of slaughtered cattle in northwestern Ethiopia. Res Vet Sci. 2008;85:522–6.
34. Kebede N, Tilahun G, Hailu A. Development and evaluation of indirect hemagglutination antibody test (IHAT) for serological diagnosis and screening of bovine cysticercosis in Ethiopia. SINET Ethiop J Sci. 2008;31:135–40.
35. Kebede N, Tilahun G, Hailu A. Current status of bovine cysticercosis of slaughtered cattle in Addis Ababa abattoir, Ethiopia. Trop Anim Health Prod. 2009;41:291–4.
36. Kinfe G, Admassu B, Getaneh G, Haile B. Study on the prevalence of bovine cysticercosis in Gondar Elfora abattoir, Gondar, Ethiopia. World J Biol Med Sci. 2016;3:14–23.
37. Kumar A, Berhe G. Occurrence of cysticercosis in cattle of parts of Tigray region of Ethiopia. Haryana Vet. 2008;47:88–90.
38. Markos A. Prevalence of bovine cysticercosis and hydatidosis in Wolayita Soddo municipal abattoir, southern Ethiopia. MSc Thesis, Haramaya University, Haramaya, Ethiopia; 2013.
39. Megersa B, Tesfaye E, Regassa A, Abebe R, Abunna F. Bovine cysticercosis in cattle slaughtered at Jimma municipal abattoir, south-western Ethiopia: prevalence, cyst viability and its socio-economic importance. Vet World. 2010;33:257–62.
40. Mekbib B, Abesha H, Tesfaye D. Study on zoonotic metacestodes of cattle slaughtered at Bahir Dar municipal abattoir, northwest Ethiopia. Glob Vet. 2013;10:592–8.
41. Mekonnen K. Study on prevalence of *cysticercus bovis* in cattle at municipal abbatoir of Kofale district, west Arsi zone, Oromia regional state, Ethiopia. J Biol Agric Healthcare. 2017;7:61–74.
42. Mersie A. A survey of bovine cysticercosis and its public health importance in eastern Ethiopa. DVM thesis, FVM, Addis Ababa University, Debrezeit, Ethiopia;1997.
43. Moje N, Zewde D, Bacha B, Regassa A. Metacestodes in cattle slaughtered at Shashemene municipal abattoir, southern Ethiopia: prevalence, cyst viability, organ distribution and financial losses. Glob Vet. 2014;12:129–39.
44. Motbaynor A, Terefe Y. Prevalence of bovine cysticercosis in cattle slaughtered at Gondar Elfora abattoir. Am-Euras J Sci Res. 2015;10:243–7.
45. Regassa A, Abunna F, Mulugeta A, Megersa B. Major metacestodes in cattle slaughtered at Wolaita Soddo municipal abattoir, southern Ethiopia: prevalence, cyst viability, organ distribution and socioeconomic implications. Trop Anim Health Prod. 2009;41:1495–1502.
46. Tadesse A, Tolossa YH, Ayana D, Terefe G. Bovine cysticercosis and human taeniosis in south-west Shoa zone of Oromia region, Ethiopia. EthiopVet J. 2013;17:121–33.
47. Talu M. Study on the risk of human taeniasis and prevalence of bovine cysticercosis in Jimma town, southwestern Oromia. MSc Thesis, Addis Ababa University, Debre Zeit, Ethiopia; 2012.
48. Tamirat B, Tamirat H, Gebru M. Prevalence, financial impact and public health significance of *cysticercus bovis* at Bahir Dar municipal abattoir, Ethiopia. J Vet Med Anim Health. 2018;10:14–20.
49. Taresa G, Melaku A, Bogale B, Chanie M. Cyst viability, body site distribution and public health significance of bovine cysticercosis at Jimma, south west Ethiopia. Glob Vet. 2011;7:164–8.
50. Tegegne A, Hiko A, Elemo KK. Bovine cysticercosis and human taeniosis: animal-human health and economic approach with treatment trends in Kombolcha Town, Wollo, Ethiopia. Int J One Health. 2018;4:15–21.
51. Teklemariam AD, Debash W. Prevalence of *Taenia saginata*/cysticercosis and community knowledge about zoonotic cestodes in and around Batu, Ethiopia. J Vet Sci Technol. 2015; doi:10.4172/2157-7579.1000273.
52. Terefe Y, Redwan F, Zewdu E. Bovine cysticercosis and its food safety implications in Harari people’s national regional state, eastern Ethiopia. Onderstepoort J Vet Res. 2014;81:1–6.
53. Tesfay H, Assefa A. *Cysticercosis bovis* in eastern Tigray, northern Ethiopia. Int J Innov Sci Res. 2014;10:522-6.
54. Tesfaye D, Sadado T, Demissie T. Public health and economic significance of bovine cysticercosis in Wolaita Soddo, southern Ethiopia. Glob Vet. 2012;9:557–63.
55. Tesfaye H. Prevalence, public health and financial importance of bovine cysticercosis in cattle slaughtered at Debre zeit municipal abattoir, Ethiopia. J Heal Med Nurs. 2016;33:6–13.
56. Tolosa T, Tigre W, Teka G, Dorny P. Prevalence of bovine cysticercosis and hydatidosis in Jimma municipal abattoir , South. Onderstepoort J Vet Res. 2009;326:323–6.
57. Hailu Y, Taha A, Terefe G, Jibat T. Bovine cysticercosis and human taeniosis in Adama town, Oromia region, Ethiopia. J Vet Sci Technolo. 2015; doi:10.4172/2157-7579.S10-003.
58. Wondimagegnei K, Belete S. Prevalence and public health significance of *cysticercus bovis* in and around Debreberhan city. Eur J Appl Sci. 2015;7:199–208.
59. Worku A. *Cysticercus bovis* and *Taenia saginata*: prevalence, public health significance and community perception about meat borne zoonosis in three selected districts of West Shoa zone of Oromia region, Ethiopia. MSc Thesis, Addis Ababa University, Bishoftu, Ethiopia; 2014.
60. Yigizaw G, Tefera Y, Tintagu T. Prevalence of *cysticercus bovis* at Dessie municipal abattoir, north east Ethiopia. Abyss J Sci Technol. 2017;2:25–9.
61. Yimer A, Gebrmedehan BM. Bovine cysticercosis and hospital based retrospective survey of human taeniasis in and around Debre Brihan city, central Ethiopia. Biol Med. 2019; doi:10.4172/0974-8369.1000455.
62. Hailemariam Z, Nakao M, Menkir S, Lavikainen A, Iwaki T, Yanagida T, et al. Molecular identification of species of *Taenia* causing bovine cysticercosis in Ethiopia. J Helminthol. 2014;88:376–80.
63. Tesfaye D, Fekede D, Tigre W, Regassa A, Fekadu A. Perception of the public on the common zoonotic diseases in Jimma, Southwestern Ethiopia. Int J Med Med Sci. 2013;5:279–85.
64. Assefa A, Tesfay H. Major causes of organ condemnation and economic loss in cattle slaughtered at Adigrat municipal abattoir, northern Ethiopia. Vet World. 2013;6:734–8.
65. Belina D, Melese M. Pathological lesion survey and financial losses associated with organs and carcass condemnation in cattle slaughtered at selected abattoirs in Ethiopia. Indian J Vet Pathol. 2017;41:1–11.
66. Edo JJ, Pal M, Rahman MT. Investigation into major causes of organs condemnation in bovine slaughtered at Adama municipal abattoir and their economic importance. Haryana Vet. 2014; 53:139-43.
67. Jemal D, Kebede B. The study of major parasitic causes of organ condemnation and financial losses in cattle slaughtered at Hawassa municipal abattoir, Ethiopia. Cogent Food Agric. 2016;2: 1201183.
68. Maseresha S, Shibbiru T, Tadesse F. Major causes of organ condemnation and associated financial loss in cattle slaughtered at Hawassa municipal abattoir, Ethiopia. J Vet Med Anim Health. 2016;8:150–6.
69. Mesele G, Guadu T, Bogale B, Chanie M. Pathological conditions causing organ and carcass condemnation and their financial losses in cattle slaughtered in Gondar, northwest Ethiopia. African J Basic Appl Sci. 2012;4:200–8.
70. Moje N, Abdeta D, Kebede S, Terfa T, Desissa F, Regassa A. Major causes of organs and carcass condemnation in cattle slaughtered at Nekemte municipality abattoir, east Wollega Ethiopia. Glob Vet. 2014;13:278-84.
71. Shiferaw S, Kumar A, Amssalu K. Organs condemnation and economic loss at Mekelle municipal. Haryana Vet. 2009;48:17–22.
72. Tefera Y, Mesfin Z, Muleta W. Major causes and abnormalities of organ condemnation and financial loss in cattle slaughtered at Dessie municipal abattior north eastern Ethiopia. J Vet Med Anim Health. 2016;8:56–63.
73. Mummed YY, Webb EC. Causes of beef carcass and organ condemnations in Ethiopia. Asian J Anim Vet Adv. 2015;10:147–60.
74. Efrem L, Serda B, Sibhat B, Hirpa E. Causes of organ condemnation, its public health and financial significance in Nekemte municipal abattoir, Wollega, western Ethiopia. J Vet Med Anim Health. 2015;7:205–14.
75. Mersie A. An overview of animal health situation in eastern Ethiopia. Proceeding of Ethiopin veterinry association; Dire Dawa, Ethiopia;1997. p. 36-46.
76. Solomon T, Alemu B. Economic loss caused by organ condemnation in cattle economic loss caused by organ condemnation in cattle slaughtered at Hawassa municipal abattoir, southern Ethiopia. J Glob Biosci. 2019;8:5966–77.
