# Supplementary figures and images for: Identification of risk areas and practices for Taenia saginata taeniosis/cysticercosis in Ethiopia: a systematic review and meta-analysis
Source: Parasit Vectors. 2020 Jul 29;13:375. doi: 10.1186/s13071-020-04222-y (PMC7391523; doi:10.1186/s13071-020-04222-y)

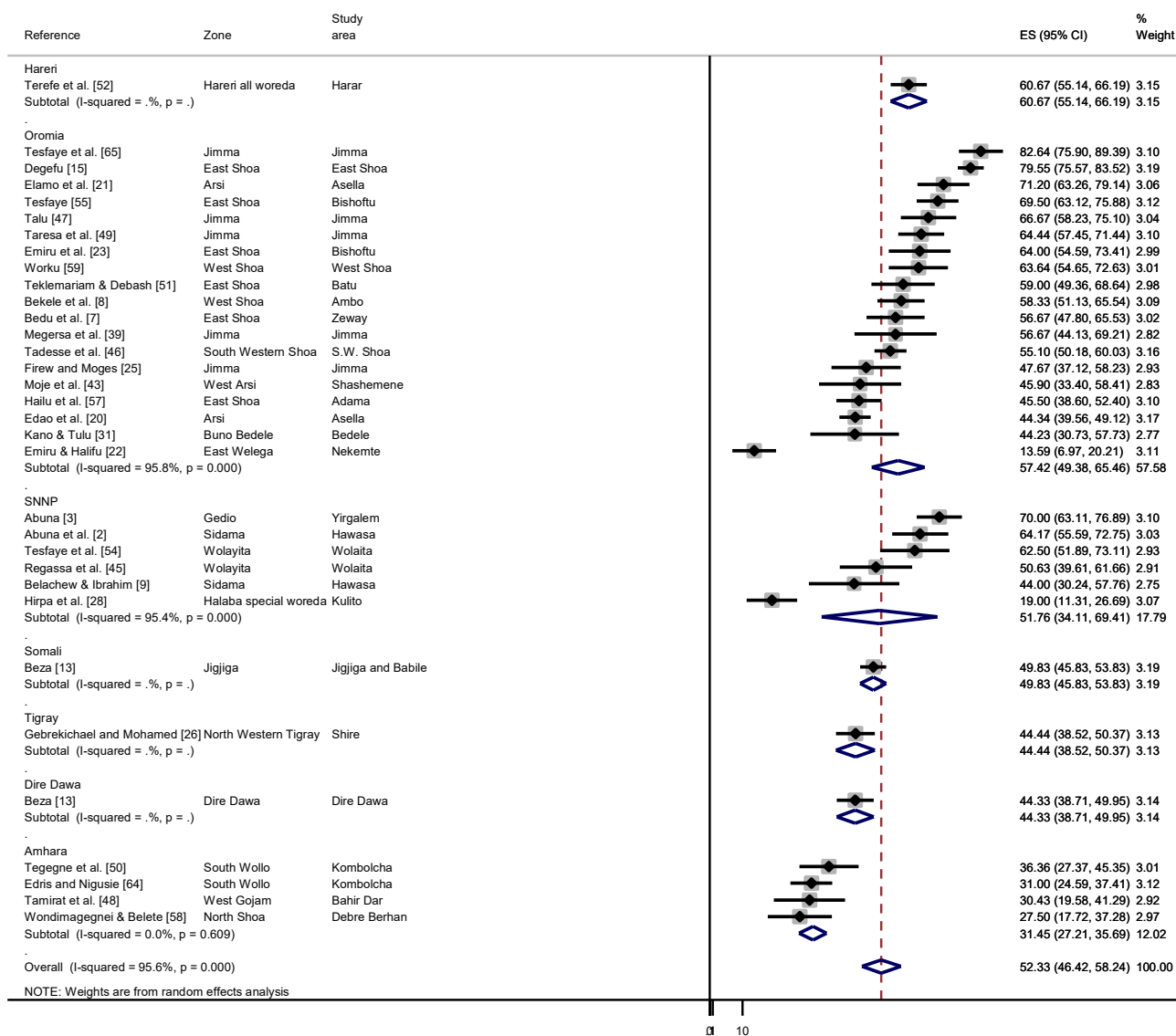

Supplement: Supplementary file 3 — Additional file 3: Figure S1. Overview of studies reporting human taeniosis (questionnaire-based diagnosis) in Ethiopia. The forest plot contains a horizontal line representing the results of each study and the length of the straight line indicates the 95% CI, the box size the weight of the study and the middle of the box the point estimate of the study. A vertical broken line is the pooled estimate and a diamond shaped box at the bottom is the CI, while the solid line shows the point of null assumption. [file 13071_2020_4222_MOESM3_ESM.pdf]
